# Transcriptomics and Metabolomics Integration Reveals Redox-Dependent Metabolic Rewiring in Breast Cancer Cells

**DOI:** 10.3390/cancers13205058

**Published:** 2021-10-09

**Authors:** Marcella Bonanomi, Noemi Salmistraro, Giulia Fiscon, Federica Conte, Paola Paci, Valentina Bravatà, Giusi Irma Forte, Tatiana Volpari, Manuela Scorza, Fabrizia Mastroianni, Stefano D’Errico, Elenio Avolio, Gennaro Piccialli, Anna Maria Colangelo, Marco Vanoni, Daniela Gaglio, Lilia Alberghina

**Affiliations:** 1 ISBE. IT/Centre of Systems Biology, 20126 Milan, Italy; marcella.bonanomi@unimib.it (M.B.); noemi.salmistraro@ibfm.cnr.it (N.S.); tvolpari@nyscf.org (T.V.); scorza@ceinge.unina.it (M.S.); fabrizia.mastroianni@unimib.it (F.M.); annamaria.colangelo@unimib.it (A.M.C.); marco.vanoni@unimib.it (M.V.); lilia.alberghina@unimib.it (L.A.); 2 Department of Biotechnology and Biosciences, University of Milano-Bicocca, 20126 Milan, Italy; 3 Institute of Molecular Bioimaging and Physiology (IBFM), National Research Council (CNR), 20054 Segrate, MI and 90015 Cefalù, PA, Italy; valentina.bravata@ibfm.cnr.it (V.B.); giusi.forte@ibfm.cnr.it (G.I.F.); 4 Institute for Systems Analysis and Computer Science “Antonio Ruberti” (IASI), National Research Council (CNR), 00185 Rome, Italy; giulia.fiscon@iasi.cnr.it (G.F.); federica.conte@iasi.cnr.it (F.C.); paola.paci@iasi.cnr.it (P.P.); 5 Fondazione per la Medicina Personalizzata, 16122 Genova, Italy; 6 Department of Computer, Control and Management Engineering, Sapienza University of Rome, 00185 Rome, Italy; 7 The New York Stem Cell Foundation Research Institute, New York, NY 10019, USA; 8 Department of Pharmacy, University of Naples “Federico II”, 80131 Naples, Italy; stefano.derrico@unina.it (S.D.); picciall@unina.it (G.P.); 9 Institute of Atmospheric Sciences and Climate (ISAC), National Research Council (CNR), 88046 Lamezia Terme, Italy; e.avolio@isac.cnr.it

**Keywords:** CtBP2, cancer metabolic rewiring, epigenetics, transcriptomics, metabolomics integration

## Abstract

**Simple Summary:**

Metabolic rewiring fuels cancer proliferation by enhanced glycolysis and the increased NADH/NAD^+^ ratio. In this study, we highlight the critical role of NADH in the epigenetic landscape mediated by CtBP2 (C-terminal binding protein 2) activation, linking metabolism to epigenetic transcriptional reprogramming. Moreover, using metabolomics and transcriptomics integration, we show that genetic and pharmacological down-regulation of CtBP2 strongly reduces cell proliferation by modulating the redox balance, nucleotide synthesis, reactive oxygen species (ROS) generation, and scavenging. Therefore, we provide evidence that metabolic rewiring plasticity regulates the crosstalk between metabolism and the transcriptional program that sustains energetic and anabolic demands in cancer cells.

**Abstract:**

Rewiring glucose metabolism toward aerobic glycolysis provides cancer cells with a rapid generation of pyruvate, ATP, and NADH, while pyruvate oxidation to lactate guarantees refueling of oxidized NAD^+^ to sustain glycolysis. CtPB2, an NADH-dependent transcriptional co-regulator, has been proposed to work as an NADH sensor, linking metabolism to epigenetic transcriptional reprogramming. By integrating metabolomics and transcriptomics in a triple-negative human breast cancer cell line, we show that genetic and pharmacological down-regulation of CtBP2 strongly reduces cell proliferation by modulating the redox balance, nucleotide synthesis, ROS generation, and scavenging. Our data highlight the critical role of NADH in controlling the oncogene-dependent crosstalk between metabolism and the epigenetically mediated transcriptional program that sustains energetic and anabolic demands in cancer cells.

## 1. Introduction

Cancer is a complex, multifactorial disease that originates and establishes due to a complex interplay of oncogenic mutations, epigenetic alterations, and metabolic rearrangements that affect cancer cells’ proliferation, survival, and differentiation. An alteration of metabolism, notably the energetic metabolism, is recognized as a cancer hallmark [1] and is a crucial component of cellular transformation [2]. Indeed, metabolism may act as an integrator of multiple intertwined regulatory layers, thus we can identify a specific metabolic footprint for each physio-pathological situation [3].

Alterations in proto-oncogenes, such as *Ras* and *MYC*, or the loss of tumor suppressors (such as *TP53* and *VHL*), promote metabolic rewiring compared to normal tissues [4,5,6,7,8,9,10]. A hyper-glycolytic phenotype (Warburg effect) occurs not only in hypoxic conditions, typical of avascular tumor mass or in the presence of mitochondrial dysfunction, but also under conditions in which the available oxygen is not sufficient to fully oxidize glucose and glutamine, as it is often the case even under normoxic conditions [11]. Cancer cells can engage glucose or glutamine metabolism as a function of the availability of one or the other, thereby developing environmental adaptation under stress conditions [12]. Besides the Warburg effect, cancer metabolic rewiring may involve elevated rates of lipid biosynthesis [13,14].

Metabolites such as ATP, acetyl-CoA (AcCoA), reactive oxygen species (ROS), and enzymes with critical roles in metabolic regulation may act on different signaling pathways controlling tumor growth [15,16,17], with the effect of an abnormal NAD^+^/NADH balance ratio [5,6]. A shift in the NAD^+^/NADH redox balance profoundly influences specific proteins that directly utilize oxidized or reduced NADH as cofactors, ligands, or substrates and can influence epigenetic regulatory events. Usually, deregulation of the redox potential is associated with these metabolic changes due to excessive ROS production by enhanced or dysfunctioning oxidative phosphorylation (OXPHOS), or due to the different usage of reducing equivalents (i.e., NADH/NADPH) in metabolic reactions, including those required for ROS detoxification [5].

The C-terminal binding proteins CtBP1 and CtBP2 are transcriptional co-regulators. They interact with DNA-binding transcription factors and recruit transcription complexes, including several chromatin-modifying proteins able to modify the target genes [18,19,20] epigenetically. As a result, CtBPs silence or activate the expression of tumor suppressor genes and proto-oncogenes. CtBP1 and CtBP2 form homo and heterodimers that are active co-transcriptional regulators. CtBP1 and CtBP2 share a highly conserved 5–6 amino acids binding motif (PXDLSK) that is able to adapt its conformation when bound to either NAD^+^ or NADH [21]. The binding affinity of CtBP is over 100-folds higher for NADH than NAD^+^, suggesting that these proteins play an essential role as metabolic sensors of the redox status, able to control epigenetic modifications that influence cellular development, function, and fate [22].

Several studies have provided evidence that CtBPs play a broad role in cancer evolution and progression by controlling gene expression of various transcriptional regulators and gene networks [23,24]. Many of these networks are associated with malignant behavior in several cell types [21]. CtBP plays a significant role in the incidence, growth, and progression of human tumors by recruitment and interaction with multiple genes involved in various processes, such as differentiation, cell proliferation, and development [21]. Indeed, global profiling of CtBP in breast cancer cells showed that many of the CtBP target genes are involved in significant cancer pathways, including the promotion of uncontrolled growth, resistance to chemotherapy, and invasion and metastasis of more aggressive cancer forms [25]. These tumors also present the worst clinical outcomes with high mortality within the first five years after diagnosis [25]. CtBP increases glutamate dehydrogenase activity and promotes glutaminolysis by repressing SIRT4 expression as a function of glucose concentration: thus, the CtBP-SIRT4-GDH may be part of the mechanism coordinating the metabolism of glucose and glutamine in cancer cells [26].

In this study, we show that down-regulation of the expression of the CtBP NADH-dependent transcriptional co-regulators reduces cell proliferation in a breast cancer cell line more than in untransformed, immortalized breast cell lines, partially reverting the oncogene-dependent intertwined metabolic and transcriptional rewiring that sustains energetic and anabolic demands in breast cancer cells.

## 2. Materials and Methods

### 2.1. Cell Culture

MDA-MB231 and MCF102A cell lines were obtained from the American Type Culture Collection (ATCC) (LGC Standard, Teddington, UK). The MDA-MB231 cell line was grown in Dulbecco’s modified Eagle’s medium (DMEM) containing 4 mM of L-glutamine, supplemented with 10% fetal bovine serum. The MCF102A cell line was maintained in DMEM/F-12 containing 5% horse serum, 2.5 mM L-glutamine, 20 ng/mL EGF, 100 ng/mL cholera toxin, 0.01 mg/mL insulin, and 500 ng/mL hydrocortisone. All media were supplemented with 100 U/mL penicillin and 100 µg/mL streptomycin, and cells were incubated at 37 °C in a 5% CO_2_ incubator. All reagents for media were purchased from Life Technologies, Waltham, MA, USA.

### 2.2. Cell Proliferation Analysis

Cells were plated in 6-well plates in the appropriate normal growth medium. For proliferation curves under nutrient deprivation conditions, the culture medium was replaced after 18 h with either normal growth medium, low-glutamine medium (0.5 mM Gln), or low-glucose medium (1 mM Glc). Cells were collected and counted after 24, 48, 72, and 144 h. For dose-response curves, the culture medium was replaced after 18 h with the normal growth medium and the drugs sodium 2-hydroxyimino-3-phenylpropanoate (HIPP), sodium 2-hydrazone-3-phenylpropanoate (P4), and 4-methylthio-2-oxobutanoate (MTOB) were added at the indicated concentrations. Cells were counted after 24 h. For time-course experiments, the culture medium was replaced after 18 h with the normal growth medium and drugs were added (HIPP 400 μM–P4 300 µM). Cells were collected and counted after 24, 48, 72, and 144 h.

### 2.3. Metabolites Quantification in the Media Samples

Absolute quantification of glucose, lactate, glutamine, and glutamate in spent media was determined enzymatically using the YSI2950 bioanalyzer (YSI Incorporated, Yellow Springs, OH, USA). Media collected from experiments were thawed and centrifuged at 2000× *g* rpm for 5 min prior to analysis. The YSI bioanalyzer employed enzyme-based biosensors for measuring glucose, lactate, glutamate, and glutamine concentrations. The biosensors used oxidases-containing membranes for oxidizing substrates, releasing hydrogen peroxide. The hydrogen peroxide was detected amperometrically at a platinum electrode surface. The current flow at the electrode was directly proportional to the hydrogen peroxide concentration and hence to the substrate concentration. Glucose, lactate, glutamine, and glutamate standard solutions were used to calibrate the instrument. Glucose and glutamine consumption as well as lactate and glutamate release were calculated as follows: consumption = mmol/L of compound in fresh complete media–mmol/L of compound in cultured media and release = mmol/L of compound in cultured media–mmol/L of compound in fresh complete media. The rates were reported as mmol/L per 10^5^ cells.

### 2.4. Metabolite Extraction from Cell Culture

For untargeted experiments, cells were plated in 6-well plates with normal growth medium; the culture medium was replaced after 18 h with complete fresh medium in the presence or absence of treatments and cells were then incubated for 48 h. For labeling experiments, cells were incubated for 48 h in fresh media supplemented with 25 mM U-^13^C_6_-Glc for MDA-MB231 or 17.5 mM for MCF102A (purchased by Cambridge Isotope Laboratories, Tewksbury, MA, USA) in the presence or absence of treatments.

For metabolites extraction for LC-MS analysis, cells were quickly rinsed with NaCl 0.9% and quenched with 500 µL ice-cold 70:30 acetonitrile:water. Plates were placed at −80 °C for 10 min, collected by scraping, sonicated twice for 5 s for five pulses at 70% power, and then centrifuged at 12,000× *g* for 10 min at 4 °C. The supernatant was collected in a glass insert and evaporated under airflow at 37 °C. Samples were then resuspended with 150 μL of H_2_O before analyses.

### 2.5. LC-MS Metabolic Profiling

LC separation was performed using an Agilent 1290 Infinity UHPLC system and an InfintyLab Poroshell 120 PFP column (2.1 × 100 mm, 2.7 μm; Agilent Technologies, Santa Clara, CA, USA). The injection volume was 15 μL at a flow rate of 0.2 mL/min with column temperature set at 35 °C. Both mobile phase A (100% water) and B (100% acetonitrile) contained 0.1% formic acid. LC gradient conditions were: 0 min: 100% A; 2 min: 100% A; 4 min: 99% A; 10 min: 98% A; 11 min: 70% A; 15 min: 70% A; and 16 min: 100% A with 5 min of post-run. MS detection was performed using an Agilent 6550 iFunnel Q-TOF mass spectrometer with the Dual JetStream (Agilent Technologies, Santa Clara, CA, USA) source operating in negative ionization mode. MS parameters were: gas temperature: 285 °C; gas flow: 14 L/min; nebulizer pressure: 45 psig; sheath gas temperature: 330 °C; sheath gas flow: 12 L/min; VCap: 3700 V; fragment: 175 V; skimmer: 65 V; and octopole RF: 750 V. Active reference mass correction was done through a second nebulizer using the reference solution (*m/z* 112.9855 and 1033.9881) dissolved in the mobile phase 2-propanol-acetonitrile-water (70:20:10 *v/v*). Data were acquired from *m/z* 60–1050. Data analysis and isotopic natural abundance correction were performed using MassHunter Profinder (version 10.0.2), which is part of the MassHunter VistaFlux software (version 1.9.0). Data preprocessing was performed using the Batch Targeted Feature Extraction algorithm and Agile 2 algorithm, and using the Batch Isotopologue Extraction algorithm for labeling data preprocessed, and both are included in the Profinder MassHunter VistaFlux software. This software assigned identities to metabolites by searching against an in-house compound database built with Agilent PCDL Manager (version B.08.00) based on the metabolite formula and its corresponding retention time (RT, File S7) with a score > 75%. In particular, batch targeted feature extraction parameters are: mass tolerance, ±10 ppm, RT, ±0.2 min, and EIC windows, ±1 min. The feature files generated containing extracted compound features and sample group information were imported into the Metaboanalyst 5.0 software for statistical analysis (File S1).

### 2.6. Seahorse Oxygen Consumption Rate

According to the manufacturer’s instructions, the cellular oxygen consumption rate (OCR) was measured by the Seahorse XF extracellular flux analyzer (Seahorse Bioscience Inc., North Billerica, MA, USA). Briefly, cells were seeded in Seahorse XF 24-well assay plates at a cell density of 20,000 cells per well in normal growth medium. After overnight attachment, the medium was washed and replaced with a pre-warmed assay medium (non-buffered DMEM supplemented with 1 mM sodium pyruvate, 25 mM glucose, and 4 mM glutamine, pH 7.4), and incubated in a non-CO_2_ incubator at 37 °C for 60 min. Basal levels of OCR were recorded, followed by the mitochondrial stress test using the inhibitor of ATP synthase oligomycin (1 μM), the uncoupler FCCP (1 μM), or the electron transport inhibitor rotenone/antimycin A (0.5 μM).

### 2.7. NADH/NAD^+^ Quantification

According to the manufacturer’s protocol, total cellular NADH and NAD+ levels were measured using the NAD^+^/NADH Quantification Kit (BioVision Inc, Milpitas, CA, USA). Briefly, 200,000 cells were extracted by homogenization in NADH/NAD extraction buffer. Samples were then directly used to detect total NAD. One aliquot was heated at 60 °C for 30 min to clear all NAD^+^ species and detect NADH. Colorimetric measurements were performed at OD 450 nm every 15 min up to 2 h using a Cary 60 ultraviolet-visible spectrophotometer (Agilent Technologies, Santa Clara, CA, USA). Quantitation was based on an NADH standard curve.

### 2.8. ROS Levels Measurement

ROS levels were measured using the DCFDA Cellular ROS Detection Assay Kit (Abcam, Cambridge, UK). Cells were harvested and stained with 20 μM 2′,7′–dichlorofluorescin diacetate (DCFDA) for 30 min at 37 °C. After that, cells were washed and fluorescence was measured at excitation/emission wavelengths of 485 nm and 535 nm, respectively, using the Cary Eclipse Fluorescence Spectrophotometer (Agilent Technologies, Santa Clara, CA, USA).

### 2.9. Stable Transfection

MDA-MB231 cells were plated in a 24-well plate at 80,000 cells/well density. Culture medium was replaced after 24 h with complete fresh medium and after 1 h, cells were transfected. The transfection was performed by mixing 0.5 μg of shRNA clone (shRNA clone set against Human NM_001329.2 and a scrambled control) (GeneCopoeia, Rockville, MD, USA) diluted in serum-free high-glucose DMEM and 1.5 μL of GenjetTM Reagent (ver. II) diluted in serum-free high-glucose DMEM (SignaGen Laboratories, Frederick, MD, USA) according to the manufacturer’s instructions. Cells were subsequently cultured for an additional 18 h, after which the medium was replaced with complete medium and Puromycin selection was performed for 1 week. Transfection efficiency was determined as the percentage of cells expressing GFP using the Countess^®^ II FL Automated Cell Counter equipped with the EVOS^®^ light cubes (Thermo Fisher Scientific, Waltham, MA, USA).

### 2.10. Real-Time PCR

Total RNA was purified using the Direct-zol RNA Miniprep Plus Kit (ZymoResearch, Irvine, CA, USA) according to the manufacturer’s instructions and was reverse transcribed using the SuperScript IV- First-Strand Synthesis Kit (Thermo Fisher Scientific Waltham, MA, USA). Real-time PCR was performed in a MiniOpticon detection system (Bio-Rad, Hercules, CA, USA) using the SsoFast EvaGreen Supermix (Bio-Rad Hercules, CA, USA). Primers were purchased from Eurofins Genomics and are shown in Table S1. For the normalization, glyceraldehyde-3-phosphate dehydrogenase (GAPDH) mRNA expression was used (Table S2).

### 2.11. Western Blot Analysis

Protein extraction was performed in RIPA buffer supplemented with protease inhibitor cocktail. Western blot experiments were performed according to the standard procedure with the following antibodies: anti-CtBP1, anti-CtBP2 (BD Biosciences, Franklin Lakes, NJ, USA), anti-β-actin (Cell Signaling Technology, Danvers, MA, USA), and anti-vinculin (Sigma-Aldrich, St Louis, MO, USA). Anti-rabbit fluorescent secondary antibody (IRDye^®^ 800CW Donkey anti-Rabbit IgG, Li-Cor Biosciences, Lincoln, NE, USA) or anti-mouse fluorescent secondary antibody (IRDye^®^ 680RD Donkey anti-Mouse IgG, Li-Cor Biosciences Lincoln, NE, USA) were used. Membranes were imaged using a Li-Cor Odyssey Fc scanner and densitometry analysis was performed using Image Studio Lite software Version 5.2 (uncropped blots are provided in File S2).

### 2.12. Whole-Genome cDNA Microarray Expression Analysis

Whole-genome cDNA microarray gene expression analyses were performed as previously reported [27,28]. Briefly, cells (from all cell lines and treatment conditions) were harvested, counted, and the pellet stored immediately at −80 °C. Total RNA was extracted using Trizol and the RNeasy mini kit (Invitrogen Corporation, Waltham, MA, USA), and its concentration, purity, and RNA integrity, measured as RNA integrity number (RIN) values, were assessed using a Bioanalyzer 2100 (Agilent Technologies, Santa Clara, CA, USA). Samples were then labelled and hybridized onto Whole Human Genome 4 × 44 K microarray Gene Chips (Agilent Technologies) containing all known genes and transcripts of the entire human genome according to the Agilent Two-Color Microarray-Based Gene Expression Analysis protocol. Microarray images were acquired with a DNA Microarray Scanner with Sure Scan High-Resolution Technology (Agilent Technologies). Background correction and normalization, as well as statistical data analyses of gene expression profiles (GEPs), were performed using Feature Extraction (version 9.5) and GeneSpring GX (version 13.0) software (Agilent Technologies, Santa Clara, CA, USA). Data presented in this work have been deposited in the National Center for Biotechnology Information Gene Expression Omnibus (GEO) (https://www.ncbi.nlm.nih.gov/geo/, accessed on 23 June 2021) and are accessible through GEO Series accession numbers (GSE178748). Microarray data are available in compliance with Minimum Information about a Microarray Experiment (MIAME) standards.

### 2.13. Transcriptomic Analysis

Expression data from high-throughput RNA-sequencing of invasive breast carcinoma were downloaded from The Cancer Genome Atlas (TCGA) data portal [29]. High-throughput RNA-sequencing data corresponded to level 3 data (i.e., normalized expression data) from RNASeq Version 2, created using MapSplice to conduct the alignment and RSEM to perform both the quantification and normalization. For the transcriptomic analysis of CtBP2 and CtBP2 target genes, we retained 103 tumors and 103 matched-normal samples (i.e., the tissue adjacent to the tumor and taken from the same patient). Differential expression analysis for microarray data of shCtBP2, HIPP, and P4-treated cells vs. control cells was performed through the following steps. For each dataset, data were processed by applying a logarithmic (log2) transformation of the expression values and by conducting a preprocessing analysis to remove (i) genes with too many zeros (i.e., not available values) among the samples and (ii) genes with too little variability, measured in terms of the InterQuartile Range (IQR). IQR measures data variability (dispersion) around the median, which is equal to the difference between the 75th and 25th percentiles of the data distribution. Thus, the smaller the IQR value, the less the data are scattered around the median. Those genes with a number of zeros greater than 75% of the total number of samples and an IQR value smaller than the 11th percentile of the IQR distribution (corresponding to those genes less scattered around the median) were filtered out. To further filter out genes that, on average, have an insufficiently large change in expression levels between the two understudy conditions (treated vs. control), we computed the fold-change as the logarithm of the ratio between the average expression of treated cells (i.e., shCtBP2, HIPP, and P4) and the average expression of control cells. We removed those genes falling behind a chosen fold-change threshold in absolute values. In particular, we set a linear fold-change threshold of 1.5 for studying the shCtBP2 dataset, 2.5 for the HIPP dataset, and 1.8 for the P4 dataset. Then, to evaluate the statistical significance of gene variation, we performed the Student’s *t*-test, and adjusted the obtained *p*-values for multiple hypothesis-testing using the False Discovery Rate (FDR) [30]. In order to select statistically significant differentially expressed genes, we set a threshold of 0.05 on the adjusted *p*-values (FDR) of each dataset. Finally, we identified a total of 463 (247 up-regulated and 216 down-regulated) differentially expressed genes between shCtBP2-treated cells and control cells, 2083 differentially expressed genes (8 up-regulated and 2075 down-regulated) between HIPP and control cells, and 293 (181 up-regulated and 112 down-regulated) differentially expressed genes between P4 and control cells (File S3). All transcriptomics data are represented as hierarchical clustering and heatmaps. The expression profiles of genes are clustered according to rows (genes) and columns (samples) by using the Pearson correlation as the distance metric and the complete linkage as the clustering method.

### 2.14. Integration Analysis between Transcriptomics and Metabolomics Data

The gene–metabolite interaction network analysis was performed using the network analysis tool by Metaboanalyst 5.0 software, which allows for the exploration and visualization of interactions between functionally related metabolites and genes. The chemical and human gene associations were extracted from STITCH [31], a search tool and resource for chemical and protein interactions (https://www.metaboanalyst.ca/MetaboAnalyst/ModuleView.xhtml, module “Network Analysis”, option “Gene-Metabolite Interaction Network”, accessed on 23 April 2021). Subnetworks with at least three nodes were generated. Most associations in STITCH are based on co-mentions highlighted in PubMed Abstracts, including reactions from similar chemical structures and similar molecular activities. In this network, metabolites are represented as squares and genes are represented as circles. Furthermore, the size of the features corresponds to its node degree and the genes circle color corresponds to its betweenness centrality values, which represent the degree of centrality a node has in a network by measuring the number of the shortest paths that pass through that node.

### 2.15. Circos Plots Analysis

The circos plots were generated with CIRCOS [32] to show the identified up-regulated and down-regulated genes and pathways.

### 2.16. Chemical Synthesis

#### 2.16.1. Synthesis of Sodium 2-Hydrazone-3-phenylpropanoate (P4)

To a solution of sodium phenylpyruvate (166 mg, 1.0 mmol) in H_2_O (2 mL), NH_2_NH_2_ (64 mg, 2.0 mmol) was added and the mixture was shaken at room temperature for 16 h. The solvents were evaporated under reduced pressure and the crude was purified by high-performance liquid chromatography (HPLC) using a Jasco UP-2075 Plus pump (Jasco Corporation, Tokyo, JP) equipped with a Jasco UV-2075 Plus UV detector (set at λ = 254 nm) and a Purospher Star 250 × 10 mm C-18 reversed-phase column (particle size: 5 µm) (MerckMillipore, Burlington, MA, USA) eluted with a linear gradient of CH_3_CN in H_2_O (from 0 to 50% in 60 min, flow 3.0 mL/min). The two prominent peaks at tR = 13.8 and 14.6 min were separately collected and evaporated. The compound with higher tR converted entirely in the other with lower tR in 24 h. The pure HIPP (140 mg, 70% yield) was recovered as an amorphous solid. The spectroscopic data agreed with that reported by Korwar, S. et al. [33].

#### 2.16.2. Synthesis of Sodium 2-Hydroxyimino-3-phenylpropanoate (HIPP)

To an ice-cooled solution of sodium phenylpyruvate (166 mg, 1.0 mmol) in H_2_O (2 mL), NaOH (80 mg, 2.0 mmol) and NH_2_OH·*HCl (140 mg, 2.0 mmol) were added, and the mixture was shaken at 0 °C for 30 min. Then, the cold bath was removed and the reaction was left at room temperature for an additional 6 h. The solvents were evaporated under reduced pressure and the crude was purified by high-performance liquid chromatography (HPLC) using a Jasco UP-2075 Plus pump equipped with a Jasco UV-2075 Plus UV detector (set at λ = 254 nm) and a Purospher Star 250 × 10 mm C-18 reversed-phase column (particle size: 5 µm) eluted with a linear gradient of CH_3_CN in H_2_O (from 0 to 50% in 60 min, flow 3.0 mL/min). The fractions containing the title compound (tR = 14.5 min.) were collected and evaporated, producing the pure HIPP (120 mg, 60% yield) as an amorphous solid. The spectroscopic data agreed with that reported by Knapp, S. et al. [34].

#### 2.16.3. Studies of Stability of P4 and HIPP

The 0.1 mM solutions of P4 and HIPP were prepared in a 10 mM phosphate buffer (pH = 7.3) and incubated for 24 h at 37 °C. The stability of P4 and HIPP was monitored after 24 h through HPLC using a Jasco UP-2075 Plus pump equipped with a Jasco UV-2075 Plus UV detector (set at λ = 254 nm) and a Purospher Star 250 × 10 mm C-18 reversed-phase column (particle size: 5 µm) eluted with a linear gradient of CH_3_CN in H_2_O (from 0 to 50% in 60 min, flow 3.0 mL/min).

### 2.17. Statistical Analysis

All experiments were performed at least in triplicates. Data are presented as mean ± SD. Observed differences were tested for significance with the Student’s *t*-test (*p*-value ** < 0.005; * < 0.01). Untargeted metabolomics statistical analysis was performed by Metaboanalyst 5.0 software. Data filtering was performed using the interquartile range (IQR) and normalization by a pooled sample from the control group following log transformation and autoscaling, and then the univariate *t*-test analysis setting FDR-adjusted the *p*-value threshold to 0.05 (File S4).

## 3. Results

**Figure 1:**
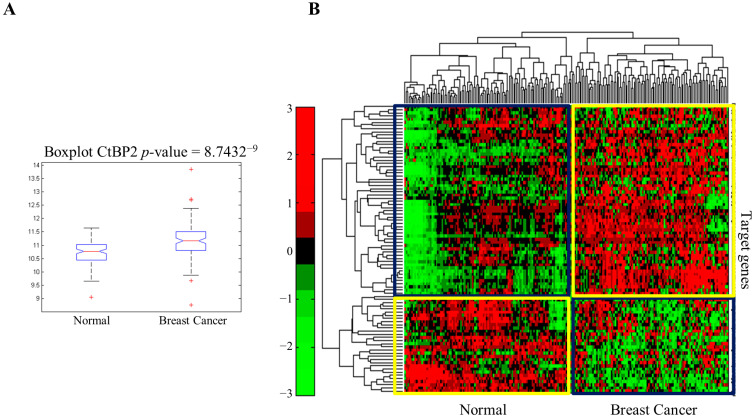

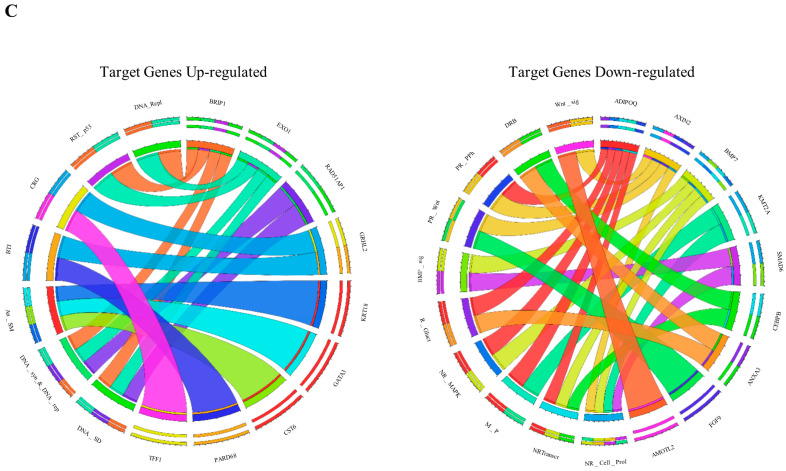
Transcriptomics profiling of human breast cancer. (**A**) Boxplot of CtBP2 expression level (logarithmic scale) in breast invasive carcinoma samples and matched normal samples retrieved from TCGA. (**B**) Hierarchical clustering and heatmap for CtBP2 target genes in invasive breast carcinoma compared to normal samples. The colors represent different expression levels that increase from green to red. (**C**) Circle spots show the most up-regulated (left panel) and downregulated (right panel) genes, and the cognate enriched pathways in human breast tissues. Up-circle pathways abbreviations: DNA SD (strand displacement); DNA syn and DNA rep (DNA synthesis involved in DNA repair); an SM (anatomical structure morphogenesis); BJT (bicellular tight junction assembly); CRG (cancer-related genes); RST p53 (regulation of signal transduction by p53 class mediator); and DNA Reply (DNA replication). Down-circle pathway abbreviations: NR Cell Prol (negative regulation of cell proliferation); MP (membrane depolarization); NR MAPK (negative regulation of MAP kinase activity); NRTranscr (negative regulation of transcription, DNA-templated); R Gluck (glucocorticoid response); BMP sig (BMP-signaling pathway); PR Wnt (positive regulation of canonical Wnt-signaling pathway); PR PPh (positive regulation of protein phosphorylation); DRB (defense response to bacterium); and Wnt sig (Wnt-signaling pathway).

CtBP1 and CtBP2 homo and hetero-dimerize in the presence of NADH to recruit various chromatin-modifying complexes that epigenetically control genes involved in animal cell development [24]. RNA-sequencing data retrieved from TCGA for 103 invasive breast adenocarcinomas and 103 matched normal samples showed that CtBP2 expression is higher in breast cancer than in normal tissues, consistent with previous data (Figure 1A). Hierarchical clustering of CtBP2 target genes highlighted a distinctive transcriptomic profiling of breast cancer samples compared to normal tissues (Figure 1B). The most significantly up-regulated CtBP2 target genes included genes whose products are involved in the positive regulation of proliferation, DNA synthesis/replication/repair mechanisms, signal transduction, and motility (Figure 1C, left panel). Significant down-regulated CtBP2 target genes included those involved in the negative regulation of cell proliferation, covering signal transduction such as MAPK, Wnt, post-translational modification, and negative regulation of transcription (Figure 1C, right panel).

### 3.1. CtBP and Metabolic Rewiring of MDA-MB231 Triple-Negative Breast Tumor Cells

**Figure 2:**
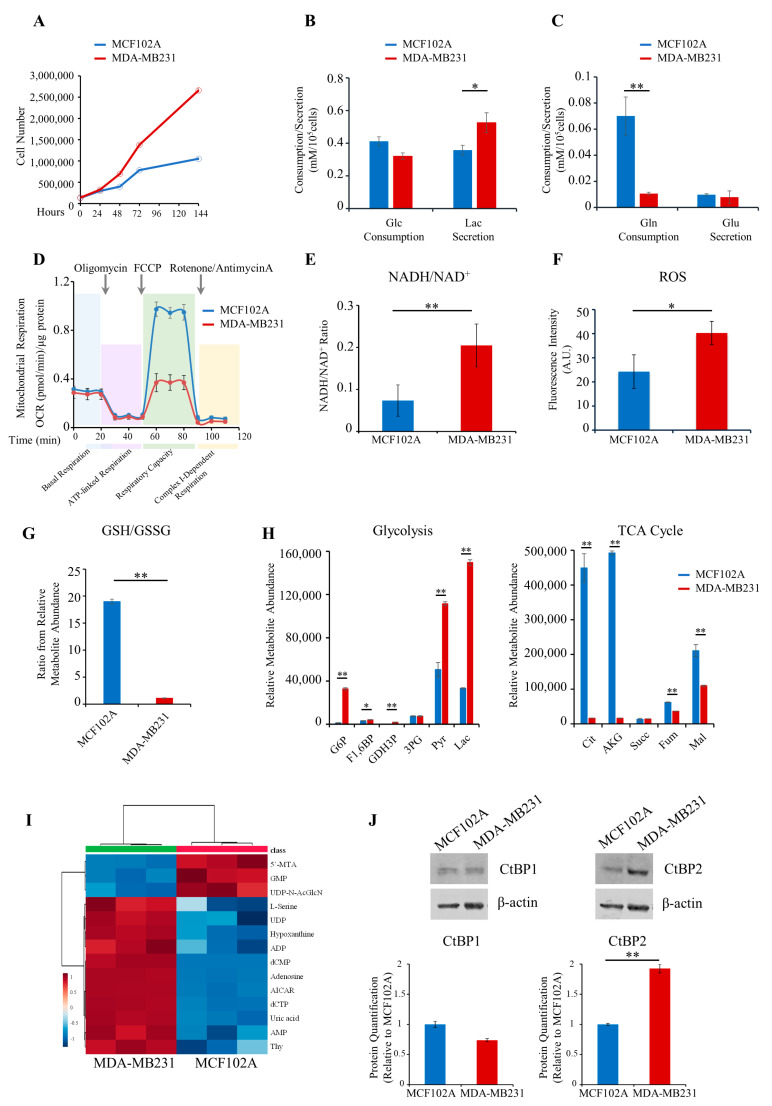
Metabolic phenotype characterization of the MDA-MB231 triple-negative breast cancer cell line. (**A**) Proliferation curves of MCF102A (–) and MDA-MB231 (–) cells. Cells were grown in 6-well plates in the appropriate growth medium. Cells were collected and counted at the indicated time points. (**B**,**C**) Extracellular uptake of Glc/Gln and secretion of Lac/Glu in MCF102A (–) and MDA-MB231 (–) cells grown for 48 h determined enzymatically using YSI2950 bioanalyzer. (**D**) Mitochondrial respiration reflected by OCR levels in MCF102A (–) and MDA-MB231 (–) cells under basal conditions or following the addition of the indicated drugs (*n* = 5). (**E**) NADH/NAD^+^ ratio in MCF102A (–) and MDA-MB231 (–) cells obtained by colorimetric assay (*n* = 9). (**F)** Intracellular ROS levels in MCF102A (–) and MDA-MB231 (–) cells measured by DCFDA staining. (**G**) GSH/GSSG ratio in MCF102A (–) and MDA-MB231 (–) cells based on relative abundance obtained by LC-MS analysis. (**H**) Relative metabolites abundance of the glycolysis (left panel) and TCA cycle (right panel) pathways in MCF102A (–) and MDA-MB231 (–) cells. (**I**) Untargeted metabolic profiling of MDA-MB231 and MCF102A cell lines grown in standard growth conditions. Hierarchical clustering heatmaps, obtained by Metaboanalyst 5.0, show significantly different intracellular metabolites by LC-MS. (**J**) Representative images of the western blot analysis of MCF102A and MDA-MB231 reporting CtBP1, CtBP2, and β-actin expression (upper panels) (relative uncropped western blot and densitometry signals can be found in File S2A). Relative densitometry analyses of CtBP1 and CtBP2 expression in the MDA-MB231 cell line compared to MCF102A (lower panels). Error bars indicate SD (*n* = 3, unless otherwise noted). ** *p*-value < 0.005; * *p*-value < 0.01.

Consistent with previous findings, triple-negative breast cancer MDA-MB231 cells showed enhanced proliferation, glucose and glutamine dependence, increased lactate, and decreased glutamine consumption than the normal MCF102A cell line (Figure 2A–C and Figure S1A). In addition, MDA-MB231 cells showed decreased maximal respiratory capacity, a higher NADH/NAD^+^ ratio, increased ROS levels, and a lower GSH/GSSG ratio than normal cells (Figure 2D–G). These observations prompted us to select these cells as a model system for further detailed metabolic analysis. Metabolomics analysis on MDA-MB231 confirms an increased level of glycolysis and lactate fermentation, decreased levels of metabolites involved in the TCA cycle, and increased levels of metabolites involved in nucleotide metabolism (Figure 2H,I).

### 3.2. Metabolomics and Transcriptomics Integration Show Metabolism/Epigenetics Networking

**Figure 3:**
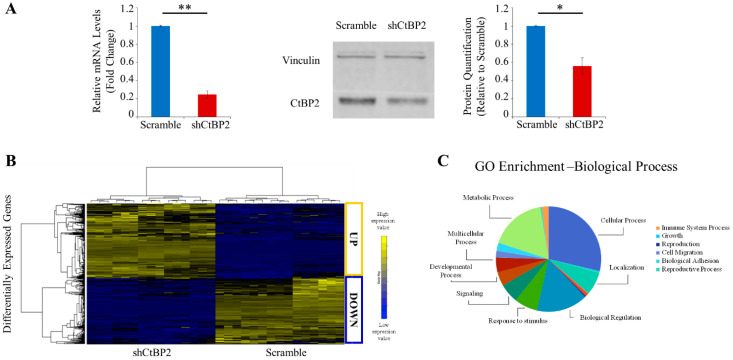

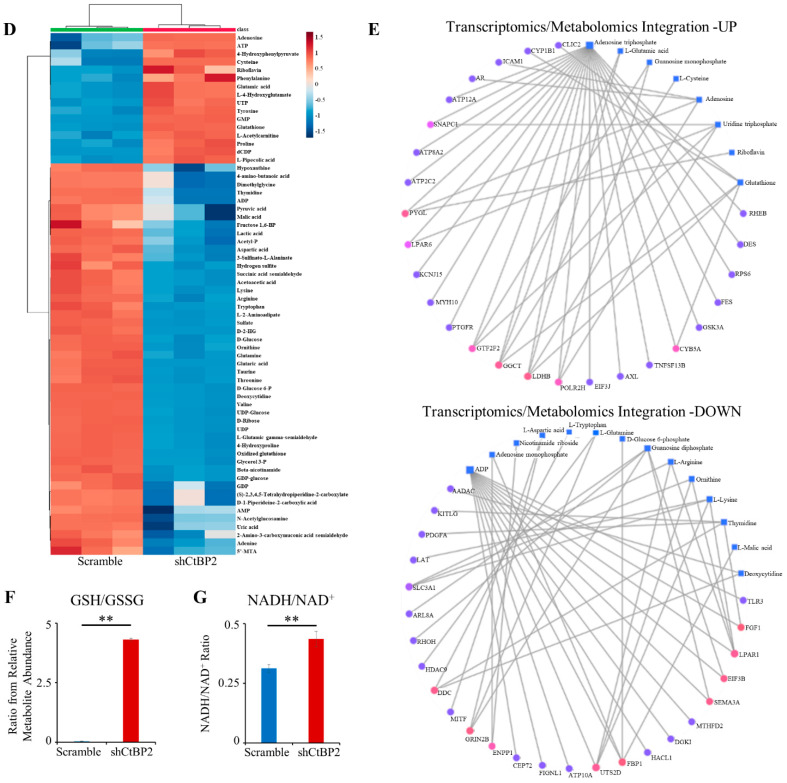
Transcriptomics/metabolomics integration of the shCtBP2 MDA-MB231 cell line. (**A**) mRNA levels expressed as a fold change of CtBP2 in shCtBP2 relative to scramble in MDA-MB231 cells, normalized for GAPDH mRNA expression (left panel); representative image of western blot analysis of MDA-MB231 cells, reporting CtBP2 and vinculin expression in shCtBP2 compared to scramble cells (middle panel); and relative densitometry analysis of CtBP2 expression in shCtBP2 relative to scramble cells (right panel) (relative uncropped western blot and densitometry signals can be found in File S2B). (**B**) Hierarchical clustering and heatmap of differentially expressed genes in MDA-MB231 cells between scramble and shCtBP2. The colors represent different expression levels that increase from blue to yellow. (**C**) Gene ontology enrichment analysis of the biological process of differentially expressed genes between MDA-MB231 scramble and shCtBP2 represented as a pie chart. (**D**) Untargeted metabolic profiling of MDA-MB231 scramble and shCtBP2 cells grown under standard growth conditions. Hierarchical clustering heatmap, obtained by Metaboanalyst 5.0, shows significantly different intracellular metabolites by LC-MS. (**E**) Gene–metabolite interaction networks of up (upper panel) and down (lower panel)-regulated genes, and metabolites in MDA-MB231 shCtBP2 cells compared to scramble cells, obtained by Metaboanalyst 5.0. Metabolites are represented as squares and genes are represented as circles. The size of the features corresponds to its node degree and the gene circle color corresponds to its betweenness centrality values (from fuchsia, which is the highest, to blue, which is the lowest). (**F**) GSH/GSSG ratio in MDA-MB231 scramble and shCtBP2 cells based on relative abundance obtained by LC-MS analysis. (**G**) NADH/NAD^+^ ratio in MDA-MB231 scramble and shCtBP2 cells obtained by colorimetric assay. Error bars indicate SD (*n* = 3). ** *p*-value < 0.005; * *p*-value < 0.01.

NADH couples glycolysis to epigenetic remodeling through the NADH-dependent dimeric family of CtBP. Therefore, we assessed the expression levels of CtBP1 and CtBP2 proteins in MDA-MB231 and MCF102A (Figure 2J). Western blotting analysis results indicate no significant difference in the expression level of CtBP1 between MDA-MB231 and MCF102A, while expression of CtBP2 was significantly (2-fold) higher in cancer cells than in MCF102A (Figure 2J). We next performed CtBP2-silencing in MDA-MB231 cancer cells by stable RNA interference of CtBP2 (shCtBP2 cells; Figure S1B). CtBP2-silencing resulted in a significant decrease (76%) in the abundance of CtBP2 mRNA, as well as a significant reduction of protein levels compared to scramble (Figure 3A). Differential expression analysis (see Methods, Section 2.13) revealed a distinct expression profile in shCtBP2 cells as compared to the scrambled control cells (Figure 3B), highlighting the CtBP2 interaction with gene networks that have broad roles in cellular homeostasis, including cellular metabolic processes, adhesion, migration, growth, signaling, environmental adaptation, and several cellular processes (Figure 3C).

Metabolomics profiling of shCtBP2 cells show decreased levels of metabolites involved in nucleotide metabolism and glucose oxidation (Figure 3D). Surprisingly, shCtBP2 cells showed significant enrichment of metabolites involved in One C metabolism (Figure S1C). The two panels in Figure 3E summarize the interactions among co-regulated metabolites and RNAs (up-regulated, top panel; down-regulated, bottom panel). RNA-metabolite interactions—derived from the STITCH database through the Joint Pathway Analysis tool in MetaboAnalyst 5.0—showed an extensive metabolism/epigenetics intertwined network between cellular energetics/nucleotides, metabolism/redox homeostasis, and CtBP2. The energetic substrates ATP and ADP are the main hubs in both panels (Figure 3E). The GSH/GSSG ratio reverses in the shCtBP2 MDA-MB231 cell line compared to the scrambled control (Figure 3F) and parental MDA-MB231 cell line (Figure 2G), suggesting that the CtBP2 knockdown elicits a partial reversion towards the normal breast phenotype. The increased NADH/NAD^+^ ratio found in shCtBP2 cells compared to the scrambled control (Figure 3G) and untransfected MDA-MB231 cells (Figure 2E) confirms that cancer metabolic rewiring by NADH is involved in the downstream CtBP2 activation and is able to alter the epigenetic landscape.

### 3.3. Low Molecular Weight CtBP2 Inhibitors Validate the Power of Metabolic Rewiring

Given the critical role of CtBP2 inhibition in the phenotype reversion of silenced MDA-MB231 and its promising role as a drug target, we sought to investigate the effect of recently described [35] small-molecule CtBP2 inhibitors: HIPP and P4 (Figure S2A). The inhibitors, designed starting from the MTOB structure [35], have a higher affinity for CtBP2 by calorimetry. Synthesis and in vitro effects on MDA-MB231 and MCF102A cells are described in the Materials and Methods Section 2.16. as well as in Figure S2, respectively.

For this purpose, we first identified the IC50 of HIPP and P4 compared to MTOB, a commercially available inhibitor of CtBP1/2 activity that has been crystalized in complexes with both proteins [36]. MTOB inhibits cell survival (Figure S2B) with an IC_50_ concentration equal to 5 mM, much higher than the IC_50_ observed for HIPP (400 μM) and P4 (300 μM) (Figure S2B). Next, we evaluated the effect of HIPP and P4 in inhibiting cell proliferation (Figure S2C), as already published for MTOB [37]. MDA-MB231 cells showed a significant reduction of cell proliferation as early as 24 h after the beginning of the HIPP and P4 treatments, while no significant effect was observed in the MCF102A normal cells (Figure S2C).

**Figure 4:**
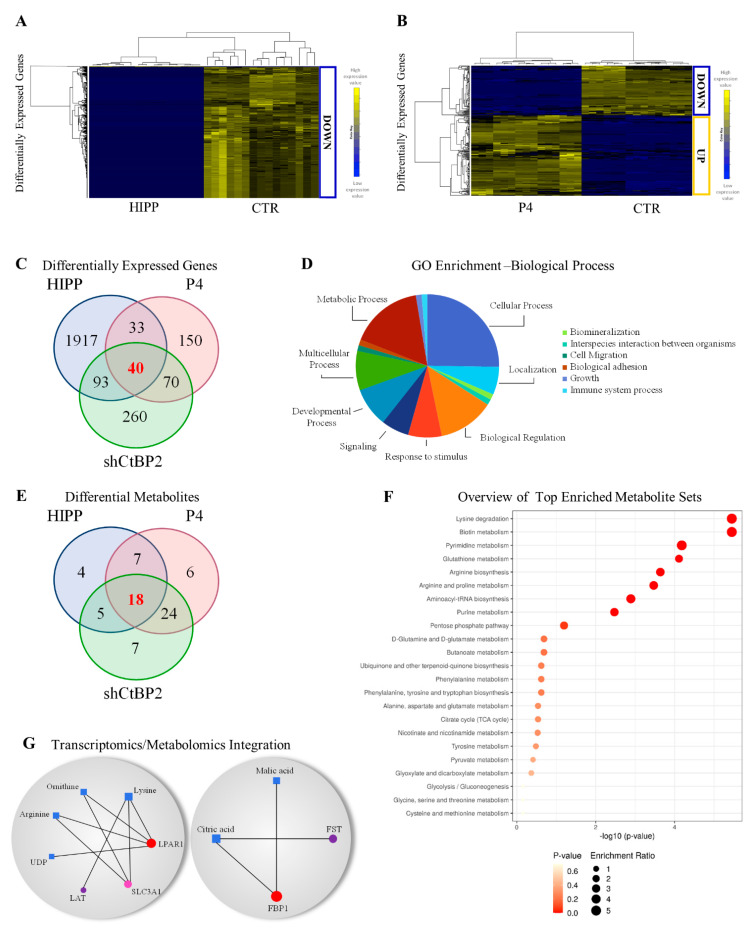
Transcriptomics/metabolomics integration of MDA-MB231 cells under HIPP and P4 chemical CtBP2 inhibitors. (**A**,**B**) Hierarchical clustering and heatmaps of differentially expressed genes in MDA-MB231 cells between the control and cells treated with HIPP (left), or the control and cells treated with P4 (right). The colors represent different expression levels that increase from blue to yellow. (**C**) Venn diagram of differentially expressed genes in HIPP, P4, and shCtBP2 compared to scramble. (**D**) Gene ontology enrichment analysis of the biological process of differentially expressed genes represented as a pie chart of the 40 common differentially expressed genes in HIPP, P4, and shCtBP2 vs. scramble cells. (**E**) Venn diagram of differential metabolites in HIPP, P4, and shCtBP2 compared to scramble. (**F**) Quantitative pathway enrichment of differential metabolites obtained by Metaboanalyst 5.0. (**G**) Gene–metabolite interaction networks of common differentially expressed genes and metabolites between HIPP, P4, and shCtBP2 compared to scramble obtained by Metaboanalyst 5.0. Metabolites are represented as squares and genes are represented as circles. The size of the features corresponds to its node degree and genes circle color corresponds to its betweenness centrality values (from red, which is the highest, to violet, which is the the lowest).

Treatment of MDA-MB231 cells for 24 h with HIPP or P4 originates distinctive transcriptional profiling compared to the untreated cells (Figure 4A,B). Forty perturbed genes (relevant in malignant tumor transformation and progression, Figure 4D) are common between shCtBP2 cells and MDA-MB231 cells treated with HIPP and P4 (see Methods Section 2.13, Figure 4C, and File S5). Quantitative mRNA expression in MDA-MB231 cells downregulated for CtBP2 and treated with both HIPP and P4 for 24 h (Figure S2D) validates the transcriptomic results for a subset of these 40 common genes. Validated down-regulated genes include NPC2 involved in the negative regulation of MAPK [38], TSPAN8 involved in tumor progression and metastasis [39], ANKRD1 mainly expressed in MDA-MB231 breast cancer cell lines and linked to treatment-resistance in breast cancer [40,41], and FST involved in metastasis suppression [42] (Figure S2D). Validated up-regulated genes include: NR3C2, AURKAIP, CDK2AP1, and ITGB1BP1 involved in epithelial-mesenchymal transition and motility [43], negative regulation of aurora kinase A [44], and G1/S transition, respectively [45] (Figure S2D).

Genetic or chemical down-regulation of CtBP2 modulates 18 common metabolites involved in nucleotide metabolism, redox metabolism, transcription, amino acids metabolism, TCA cycle, and glycolysis (Figure 4E,F). In addition, integrative omics analysis between common genes and metabolites identified a correlation between the CtBP2 and TCA cycle and amino acid metabolism by LPAR1, LAT1, Slc3A1, FST, and FBP1 genes (Figure 4G). Integration data analysis identified LPAR1 downregulation (Figure S2E) as the central hub of the network (Figure 4G) whose activity is coupled with G proteins to induce signaling for cell proliferation, cell survival, cell migration, and cytoskeletal changes, and has pro-tumorigenic, pro-migratory, and pro-metastatic effects on cancer cells [46,47,48].

**Figure 5:**
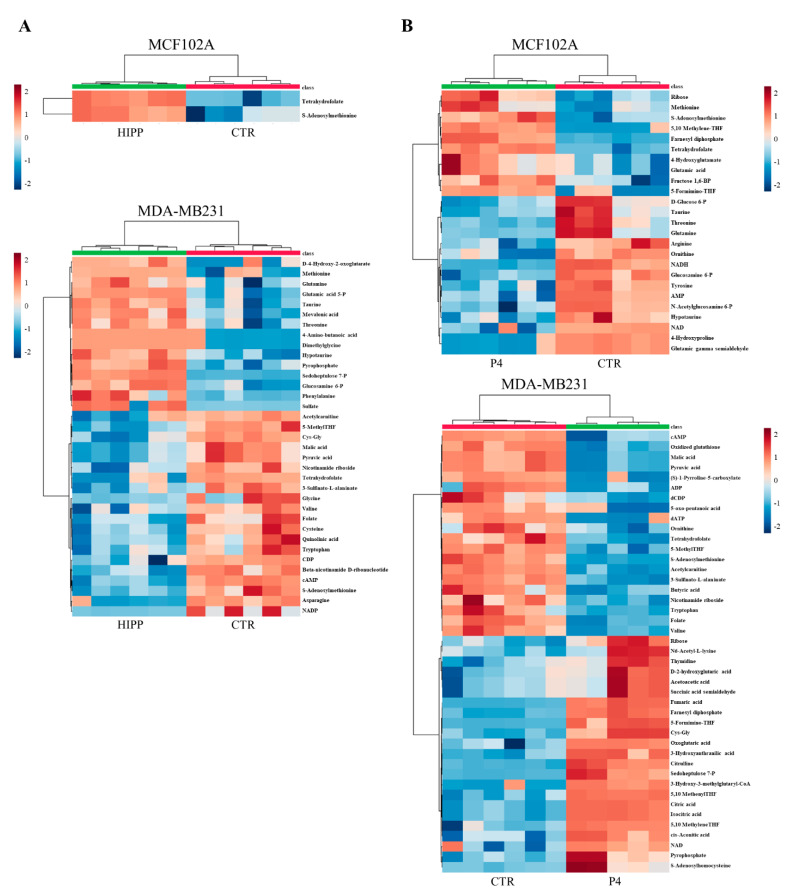
Metabolic profiling of MCF102A and MDA-MB231 under drug treatment. (**A**,**B**) Untargeted metabolic profiling of control and HIPP-treated MCF102A (upper panel) and MDA-MB231 (lower panel) cell lines (**A**), or control and P4-treated MCF102A (upper panel) and MDA-MB231 (lower panel) cell lines (**B**) grown in standard growth conditions. Hierarchical clustering heatmaps, obtained by Metaboanalyst 5.0, show significantly different intracellular metabolites detected by LC-MS.

Down-regulation in Slc3A1 and LAT (Figure S2E) involved in amino acid transport of cysteine and histidine, respectively, the latter being an essential amino acid involved in aspartate and glutamate synthesis [49], is consistent with the decreased glutathione (Figure 5B), suggesting that coordination with ASCT2 is frequently co-opted to support metabolic rewiring [50].

**Figure 6:**
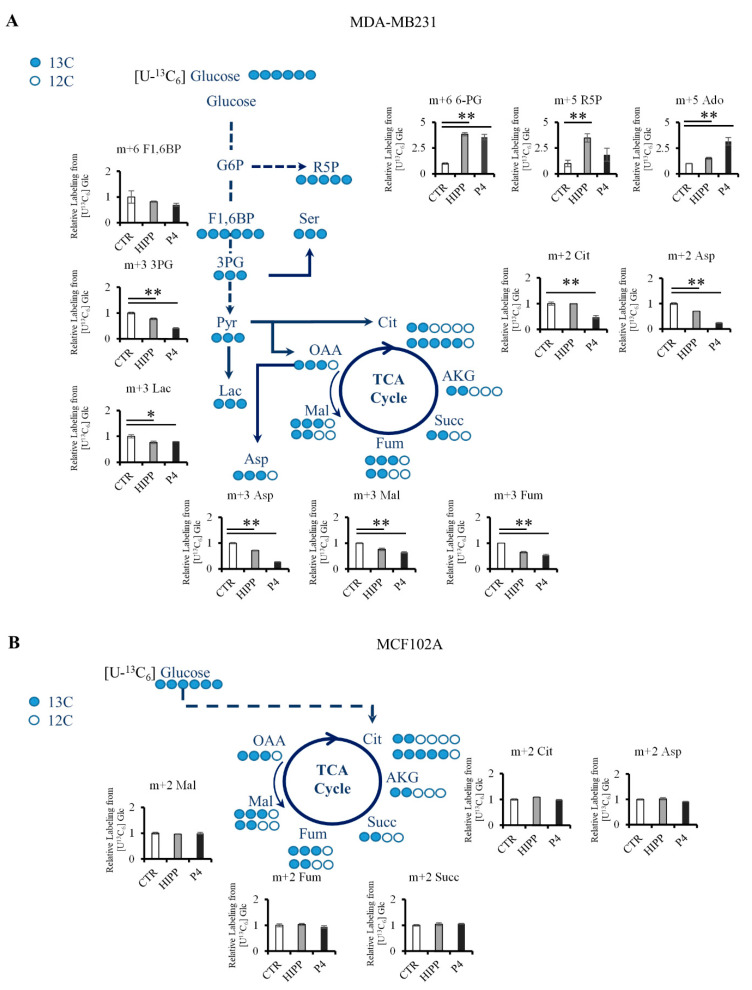
Glucose metabolic rewiring by (U-^13^C_6_) glucose labeling. (**A**,**B**) Atom transition map of (U-^13^C_6_) glucose (blue circles) used to detect metabolic changes of control or drug-treated MDA-MB231 (**A**) or MCF102A (**B**). Filled circles indicate 13C enrichment. ** *p*-value < 0.005; * *p*-value < 0.01.

Consistent with the shCtBP2 metabolomics profiling, (U-^13^C_6_) glucose isotope labeling shows decreased glucose oxidation labeling via lactate and non-canonical TCA cycles in MDA-MB231 under HIPP and P4 treatments (Figure 6A). Specifically, the absence of M2 labeling suggests a constitutive truncated TCA cycle in untreated MDA-MB231 triple-negative breast cancer cells compared to normal cells (Figure 6B). However, this truncated TCA cycle can sustain anabolic processes: through the M2 citrate branch, it sustains M2 aspartate via ATP-citrate lyase, while the M3 malate labeling (deriving from pyruvate carboxylase) sustains the TCA cycle second branch and M3 aspartate synthesis (Figure 6A). Additionally, we found a further CtBP2-independent metabolic rearrangement based on increased levels of metabolites in the pentose phosphate pathway (Figure 5A and Figure 6A) and in One C metabolism (Figure 5A,B). Moreover, metabolic profiling of MCF102A shows a significant reduced metabolic effect of HIPP treatment as compared to P4, suggesting that HIPP might be an effective treatment for tumor cells with lower side effects on normal cells (Figure 5A).

## 4. Discussion

Cancer cells show a unique metabolic phenotype compared to normal cells. Many cancer cells preferentially use aerobic glycolysis coupled to lactate rather than oxidative phosphorylation [4,51]. This rewired glucose metabolism allows cancer cells to sustain high proliferation rates, fast generation of ATP to sustain energy demands, and higher lactate fluxes to guarantee NADH/NAD^+^ redox balance [6,12]. Since glucose alone cannot provide all the building blocks required to sustain cell growth, other nutrients—such as glutamine—are required and are essential for cancer hyper-proliferation [6,11]. Glucose can also flux through the pentose phosphate pathway (PPP), which generates NADPH and purines, and the hexosamine pathway is required for glycosylation of proteins and metabolism. Recent evidence shows that cancer cells may show metabolic plasticity and switch to glucose oxidation depending on the micro-environmental context [5,52].

In this study, we use a systems metabolomics approach [53], combining metabolomic and transcriptomic profiling with metabolic labeling, to probe the response of MDA-MB231, a triple-negative breast cancer cells line that shows a high glycolytic flux and a reduced maximal respiratory ability, to genetic or pharmacological perturbation of the CtBP proteins. Transcriptomic and metabolomics data of MDA-MB231 cells whose CtBP2 expression has been silenced by shRNA show unique transcriptomic and metabolomic profiles, i.e., the downregulation of genes and metabolites strongly correlated with metabolic and cell biological processes that are associated with uncontrolled tumor growth (Figure 3).

By comparing the differential expression of the CtBP2 target genes in TCGA tissues and cell lines, we found that: (i) for the shCtBP2-treated cells, two genes (i.e., HOPX and TFF1), upregulated in TCGA cancer tissues, were downregulated following the CtBP2 inhibition, whereas only KLF8, downregulated in TCGA cancer tissues, was upregulated following the CtBP2 inhibition (File S6); (ii) for the HIPP-treated cells, 12 target genes, upregulated in TCGA cancer tissues, exhibited a downregulation following the CtBP2 inhibition (File S6); and for the P4-treated cells, two genes (i.e., COL8A1 and HDAC2), upregulated in TCGA cancer tissues, were downregulated following the CtBP2 inhibition, whereas only CEBPB, downregulated in TCGA cancer tissues, was upregulated following the CtBP2 inhibition (File S6). The deregulation of CtBP2 target genes in breast cancer samples with respect to normal tissues was also confirmed by analyzing another TCGA-independent datasets [54] available from the GEO public repository at the accession number GSE37751 (File S6 and Figure S3).

Although each of the tested CtBP perturbations has a distinct transcriptional and metabolic profile, the presence of a large subset of genes and metabolite commonly perturbed by all of the tested treatments further confirms the specificity of our results. Inhibition of CtBP2 expression or function by shRNA or by the chemical compounds HIPP and P4 strongly activates the ROS-scavenger pathways (Figure 3 and Figure 4). In particular, we observed a significant increase in the GSH/GSSG and NADH/NAD^+^ ratios in CtBP2-silenced cells compared to scrambled-treated cells (Figure 3F,G, respectively). The effect was more dramatic on the GSH/GSSG ratio that increased to almost five in the silenced cells, while remaining well below or close to 1 in the scrambled-treated or untreated cells, respectively. The NADH/NAD^+^ ratio increased from 0.2 and 0.3 (untreated and scrambled-treated, respectively) to 0.5 in the silenced cell line. Both alterations indicate that the partial inability of the silenced cell line to properly sense NADH signals an oxidative stress situation that is partially compensated by increasing the level of NADH itself and of GSH, a major cellular anti-oxidant.

Several key metabolites, notably NADH and acetylCoA, are substrates or cofactors of chromatin-modifying enzymes: as a result, metabolic rewiring and epigenetic remodeling are directly connected and reciprocally regulate each other [55,56,57]. By way of example, mutations in genes encoding isocitrate dehydrogenase 1 and 2 cause the accumulation of metabolites that affect the balance between histone and DNA methylation, in turn generating a wide deregulation of epigenetically controlled gene expression [58]. Our results are therefore in line with the role of CtBP proteins in sensing intracellular NADH through direct binding and ensuring the recruitment of chromatin-modifying complexes that reshape the transcriptional and metabolic landscape. The latter can provide cancer cells an important proliferation advantage and advantage against drug treatments developing resistance mechanisms. Tumor cells notoriously enhance metabolic pathways in order to use different carbon sources besides glucose [59]. Interestingly, metabolomic profiling identifies the activation of one-carbon metabolism as a crucial pathway in cell proliferation and survival in HIPP and P4-treated MDA-MB231 (Figure 5). HIPP and P4-treated cells, as well as shCtBP2 cells, reduce glutamine metabolism, consistent with published data indicating that CtBP promotes glutaminolysis [26]. U-^13^C_6_ glucose labeling (Figure 6) shows evidence for alternative routes of glucose oxidation, from the glycolysis/truncated TCA cycle to the pentose phosphate pathway, to sustain purine synthesis; this also represents an attempt to escape the effect of the chemical inhibition and ensure survival.

In line with literature data suggesting that CtBP proteins are expressed in a wide variety of tissues [60,61], we believe the reported observation could be extended to other breast tumors and possibly other tumors as well. The high metabolic adaptation ability of CtBP2 knockdown cells revealed in this study suggests how deeper understandings of cancer metabolism might be important in drug design and may lead to more efficient cancer therapies.

## 5. Conclusions

Our data show that cancer cells perturbed by genetic or chemical down-regulation of the CtBP1,2 proteins present significant transcriptional and metabolic plasticity. These adaptive mechanisms regulate the crosstalk between metabolism and the transcriptional program that sustains energetic and anabolic demands in cancer cells and can provide cancer cells an important advantage against drug treatments, eventually leading to the development of resistance mechanisms. Therefore, the possible insurgence of compensatory metabolic rewiring events induced by drug treatments must be considered when designing metabolism-based anti-cancer therapeutic regimens.

## Data Availability

The data presented in this study are available in the article or Supplementary Files. Transcriptomics data have been deposited in the National Center for Biotechnology Information Gene Expression Omnibus (GEO) (https://www.ncbi.nlm.nih.gov/geo/) and are accessible through GEO Series using the following Genomic Spatial Event (GSE) accession number GSE178748.

## Supplementary Materials

The following are available online at https://www.mdpi.com/article/10.3390/cancers13205058/s1, Figure S1: (A) Proliferation curves of MCF102A (**—**) (left panel) and MDA-MB231 (**—**) (right panel) cells, Figure S2: (A) Schematic representation of chemical compounds used to inhibit CtBP2, Figure S3: Venn diagrams of comparison between the CtBP2 target genes in the TCGA dataset and GEO_GSE37751 dataset, Table S1: List of primers sequences used for real-time PCR, Table S2: GAPDH mean threshold cycle in real-time PCR analysis for MDA-MB231 cells, File S1: This file contains extracted metabolite peak areas and sample group information obtained by MassHunter Profinder for all the metabolomics analysis performed, File S2: This file contains the images of uncropped blots related to Figure 2J (panel A) and Figure 3A (panel B), and relative densitometry analysis for each band performed using Image Studio Lite software, File S3: This table contains three sheets reporting the differentially expressed genes obtained for shCtBP2, HIPP, and P4, and compared to the control cells, along with their statistics (i.e., *p*-value, adjusted *p*-value (FDR), logarithmic fold-change, and up/down regulation), File S4.: This file contains the Student’s *t*-test analysis Excel sheets generated by Metaboanalyst 5.0 software for all the metabolomics analyses performed (i.e., *t*-statistic, *p*-value, logarithmic *p*-value, and adjusted *p*-value (FDR)), File S5: This table reports the results of the Venn diagram showed in Figure 4G, including the lists of the 40 common differentially expressed genes in HIPP, P4, and shCtBP2 vs. the control cells, together with the specific lists of each comparison, File S6: This file contains comparisons of the TCGA results obtained for CtBP2 target genes with those obtained from our cell line studies (scramble vs. shCtBP2, MDA-MB231 CTR vs. HIPP, and MDA-MB231 CTR vs. P4) and CtBP2 target genes’ expression in the GEO_GSE37751 dataset (i.e., *p*-value, adjusted *p*-value (FDR), logarithmic fold-change, and up/down regulation), File S7: In-house PCDL compound library used for targeted feature extraction.

## Abbreviations

3PG: 3-phosphoglycerate; 5,10 MethyleneTHF: 5,10 Methylenetetrahydrofolate; 5,10-MethenylTHF: 5,10-Methenyltetrahydrofolate; 5′-MethylTHF: 5′-Methyltetrahydrofolate; 5′-MTA: 5′-methylthioadenosine; 5-Formimino-THF: 5-Formiminotetrahydrofolate; 6-PG: 6-Phosphogluconic acid; Ado: adenosine; ADP: adenosine diphosphate; AKG: α-ketoglutaric acid; AMP: adenosine monophosphate; an SM: anatomical structure morphogenesis; Asp: aspartic acid; BMP sig: BMP-signaling pathway; BTJ: bicellular tight junction assembly; cAMP: cyclic-AMP; CDP: cytidine-diphosphate; Cit: citric acid; CRG: cancer-related genes; Cys: cysteine; D-2-HG: D-2-Hydroxyglutaric acid; dATP: deoxyadenosine-triphosphate; dCDP: deoxycytidine-diphosphate; dCMP: deoxycytidine-monophosphate; dCTP: deoxycytidine-triphosphate; DNA Repl: DNA replication; DNA SD: strand displacement; DNA syn and DNA rep: DNA synthesis involved in DNA repair; DRB: defense response to bacterium; F1,6BP: Fructose-1,6-bisphosphate; Fum: fumaric acid; G6P: glucose-6-phosphate; GDH3P: Glyceralaldehyde-3-phopsphate; GDP: Guanosine diphosphate; GFP: green fluorescent protein; Glc: glucose; Gln: glutamine; Glu: glutamic acid; Gly: glycine; GMP: guanosine monophosphate; GSH: glutathione; GSSG: oxidized glutathione; Lac: lactate; M P: membrane depolarization; Mal: malic acid; NR Cell Prol: negative regulation of cell proliferation; NR MAPK: negative regulation of MAP kinase activity; NRTranscr: negative regulation of transcription, DNA-templated; OAA: oxaloacetic acid; PR PPh: positive regulation of protein phosphorylation; PR Wnt: positive regulation of canonical Wnt-signaling pathway; Pyr: pyruvic acid; R Gluct: response to glucocorticoid; R5P: Ribose-5-phosphate; ROS: reactive oxygen species; RST p53: regulation of signal transduction by p53 class mediator; Ser: serine; Succ: succinic acid; TCA Cycle: Tricarboxylic acid cycle; Thy: thymidine; UDP: uridine diphosphate; UDP-N-AcGlcN: UDP-N-acetylglucosamine; and UTP: uridine triphosphate.
