# Supplementary material for: Transcriptomics and Metabolomics Integration Reveals Redox-Dependent Metabolic Rewiring in Breast Cancer Cells
Source: Cancers (Basel). 2021 Oct 9;13(20):5058. doi: 10.3390/cancers13205058 (PMC8534001; doi:10.3390/cancers13205058)
Supplement: Supplementary file 1 [file cancers-13-05058-s001.zip › cancers-1387848-SI/cancers-1387848-supplementary.pdf]

Supplementary Materials: Transcriptomics and Metabolomics Integration Reveals Redox-Dependent Metabolic Rewiring in Breast Cancer Cells

Marcella Bonanomi, Noemi Salmistraro, Giulia Fiscon, Federica Conte, Paola Paci, Valentina Bravatà, Giusi Irma Forte, Tatiana Volpari, Manuela Scorza, Fabrizia Mastroianni, Stefano D'Errico, Elenio Avolio, Gennaro Piccialli, Anna Maria Colangelo, Marco Vanoni, Daniela Gaglio and Lilia Alberghina

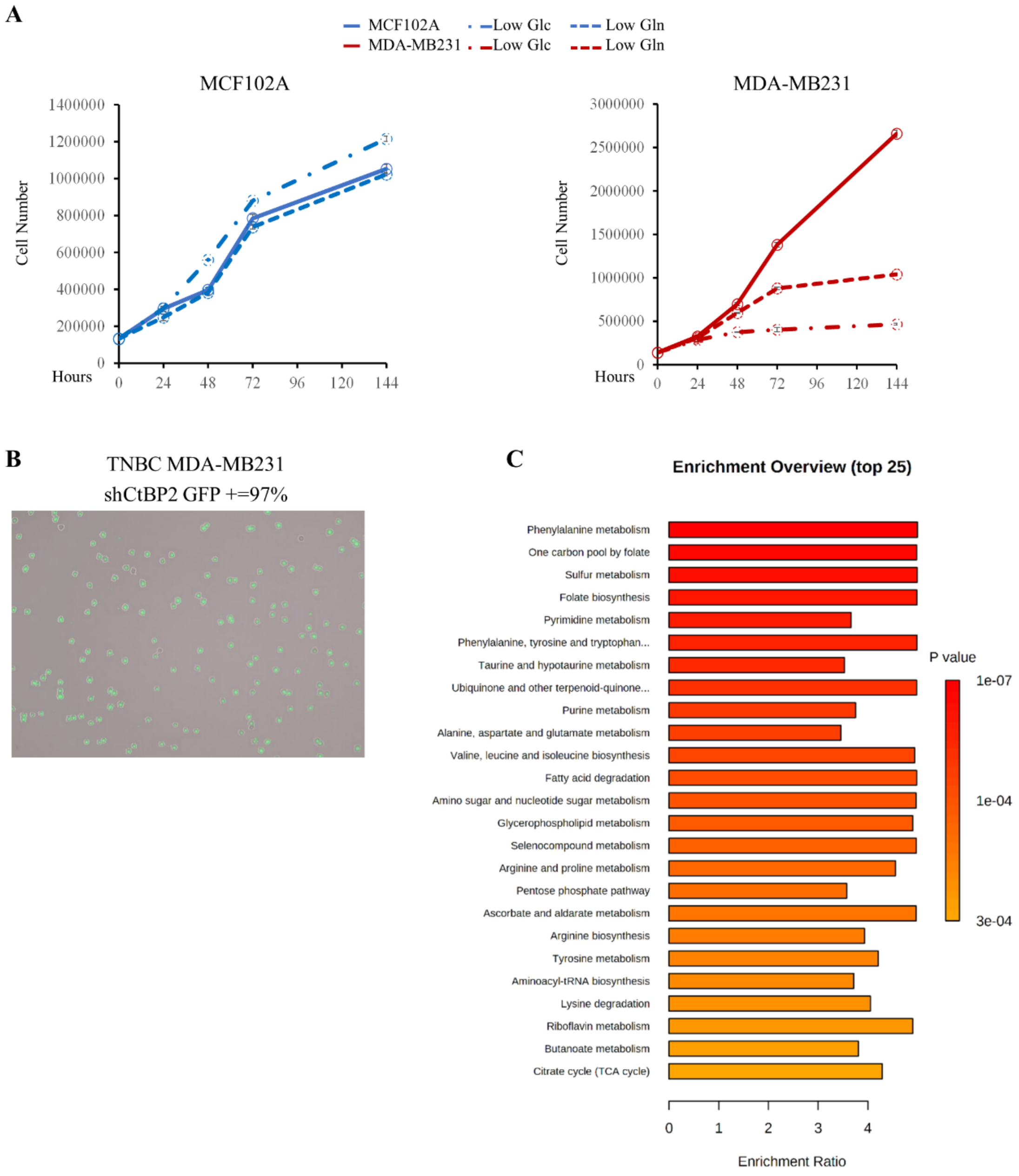

Figure S1. (**A**) Proliferation curves of MCF102A (—) (left panel) and MDA-MB231 (—) (right panel) cells. Cells were plated onto 6-well plates in standard medium. Culture medium was replaced after 18 h with standard medium (—/— Glc 25 mM), or medium containing 1 mM glucose (. —/ . —), or 0.5 mM glutamine (- - -/- - -). Cells were collected and counted at the indicated time points. Error bars indicate SD (*n* = 3). (**B**) Representative image of GFP MDA-MB231 fluorescent cells obtained by Automated Cell Counter equipped with the EVOS® light cubes to determine the transfection efficiency using shRNA clone against CtBP2. (**C**) Quantitative pathway enrichment of differential metabolites in MDA-MB231 scramble and shCtBP2 cells.

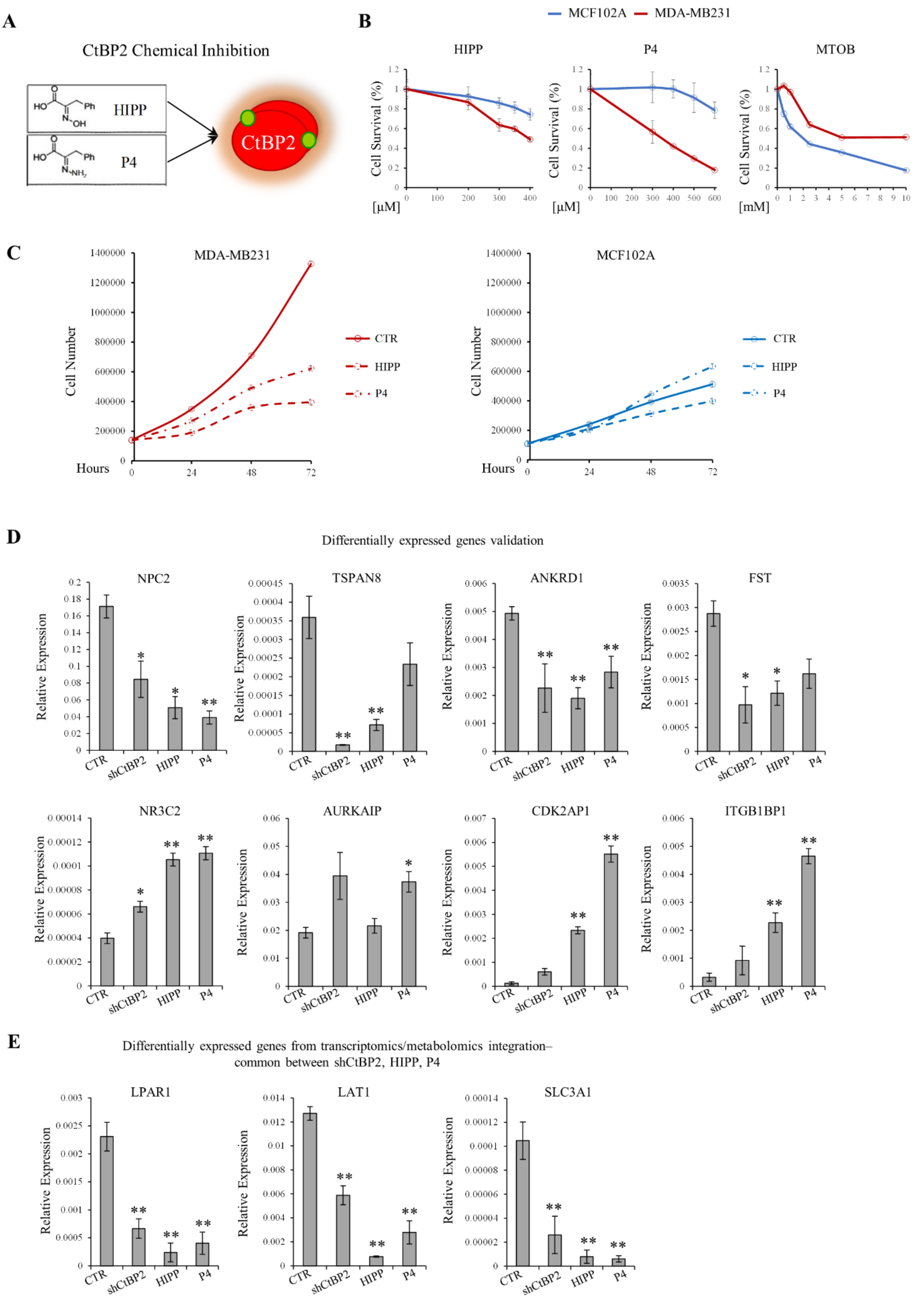

Figure S2. (**A**) Schematic representation of chemical compounds used to inhibit CtBP2. (**B**) HIPP, P4 and MTOB dose-response curves on MCF102A (▬) and MDA-MB231 (▬) cells measured as cells per well treated for 24h with 0 to 400 μM HIPP, or 0 to 600 μM P4, or 0 to 10 mM MTOB. Error bars represent SD for *n* = 3 biological replicates. (**C**) Proliferation curves of MDA-MB231 (▬) (left panel) and MCF102A (▬) (right panel) incubated with HIPP 400 μM (- - -/- - -) or P4 300 μM (. ▬/ . ▬), versus CTR (▬/▬), collected and counted at the indicated time points. (**D**) Relative expression of common genes downregulated (upper panel) and upregulated (lower panel) in shCtBP2, HIPP and P4 compared to the CTR group.

(**E**) Relative expression of common differentially expressed genes between shCtBP2, HIPP and P4 obtained by transcriptomics/metabolomics integration. *p*-value **<0.005; *<0.01.

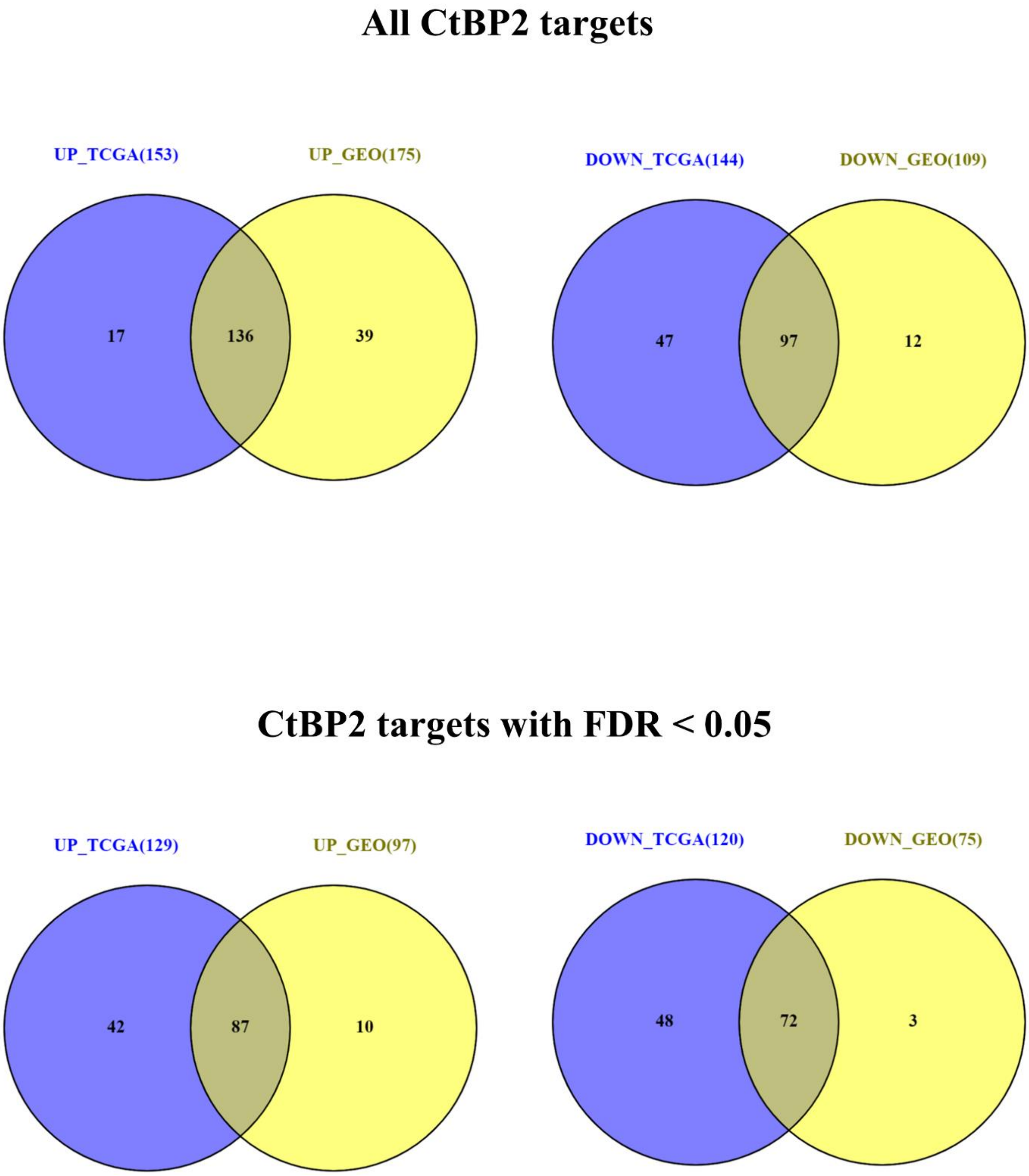

Figure S3. Venn diagrams of comparison between the CtBP2 target genes in the TCGA dataset and in GEO_GSE37751 dataset.

Table S1. List of primers sequences used for Real-time PCR.

Primers for Real-Time PCR	
**Name**	**Sequence (5′→ 3′)**
CtBP2 Forward	AACATAACCACCACCTCATCA
CtBP2 Reverse	GCTTGTGCTAAGGCTTTCTC
GAPDH Forward	TTGGTATCGTGGAAGGACTC
GAPDH Reverse	GTAGAGGCAGGGATGATGTT
NPC2 Forward	CCAGTACAGATCGTTTCTCATCTCT
NPC2 Reverse	CTGGAGGTGCTGTCAAGAGT
TSPAN8 Forward	CCCAGGAGCTATGACAAGCA
TSPAN8 Reverse	GAGATTTCTGTATCCACGGACATT
NR3C2 Forward	GAAGCCCGTGCAGTCAGTCA
NR3C2 Reverse	CTTTGGTCTCCATCGCTGCCT
AURKAIP Forward	TGAGACGCAAGCAGATCAAGT
AURKAIP Reverse	CATTTGCCCCGCAGGTAGAT
CDK2AP1 Forward	CTTACAAACCGAACTTGGCCG
CDK2AP1 Reverse	TCCCAGTTCCCTGGGTGTAG
ITGB1BP1 Forward	ACTAAGAGCAAGCAAGATGGAAAGT
ITGB1BP1 Reverse	GCATGCCTGTGCAAAACATC
ANKRD1 Forward	TAGCGCCCGAGATAAGTTTGC
ANKRD1 Reverse	GGTTCAGTCTCACCGCATCA
FST Forward	TGCCACCTGAGAAAGGCTAC
FST Reverse	CTTCACAGGACTTTGCTTTGATAC
LPAR1 Forward	ATTTCACAGCCCCAGTTCACA
LPAR1 Reverse	ACCAGCTTGCTGACTGTGTT
LAT1 Forward	CTGGATCATCCCCGTCTTCG
LAT1 Reverse	GCGTCATCACACACGTGAAC
SLC3A1 Forward	TCGCTCAAAGTCACCAATGC
SLC3A1 Reverse	GGCTGAGTCTTTTGGACATCAA

Table S2. GAPDH mean threshold cycle in Real Time-PCR analysis for MDA-MB231 cells.

	Mean Threshold Cycle (C(t))				
	Sample (MDA-MB231)				
**Target**	**CTR**	**Scramble**	**shCtBP2**	**HIPP**	**P4**
GAPDH	15.89215	15.93422	16.0576	16.02706	15.87181

File S1. This file contains extracted metabolites peak areas and sample group information obtained by MassHunter Profinder for all metabolomics analysis performed.

File S2. This file contains the images of uncropped blots related to figure 2J (panel A) and figure 3A (panel B) and relative densitometry analysis for each band performed using Image Studio Lite software.

File S3. This table contains three sheets, reporting the differentially expressed genes obtained for shCtBP2, HIPP, and P4 and compared to control cells, along with their statistics (i.e., *p*-value, adjusted *p*-value (FDR), logarithmic fold-change, up/down regulation).

File S4. This file contains Student's t-test analysis Excel sheets generated by Metaboanalyst 5.0 software for all metabolomics analysis performed (i.e., *t*-statistic, *p*-value, -logarithmic *p*-value, adjusted *p*-value (FDR)).

File S5. This table reports the results of the Venn diagram showed in Figure 4G, including the lists of the 40 common differentially expressed genes in HIPP, P4, and shCtBP2 versus control cells, together with the specific lists of each comparison.

File S6. This file contains comparisons of the TCGA results obtained for CtBP2 target genes with those obtained from our cell lines studies (scramble vs. shCtBP2, MDA-MB231 CTR vs. HIPP, MDA-MB231 CTR vs. P4) and CtBP2 target genes expression in GEO_GSE37751 dataset (i.e., *p*-value, adjusted p-value (FDR), logarithmic fold-change, up/down regulation).

File S7. This file contains in-house PCDL compound library used for targeted feature extraction, including information about metabolites formula and its corresponding retention time.

Files S1–S7. are provided separately attached as Excel and Pdf files.
