# Supplementary material for: Transcriptomics and Metabolomics Integration Reveals Redox-Dependent Metabolic Rewiring in Breast Cancer Cells
Source: Cancers (Basel). 2021 Oct 9;13(20):5058. doi: 10.3390/cancers13205058 (PMC8534001; doi:10.3390/cancers13205058)
Supplement: Supplementary file 1 [file cancers-13-05058-s001.zip › cancers-1387848-SI/File S2.pdf]

A

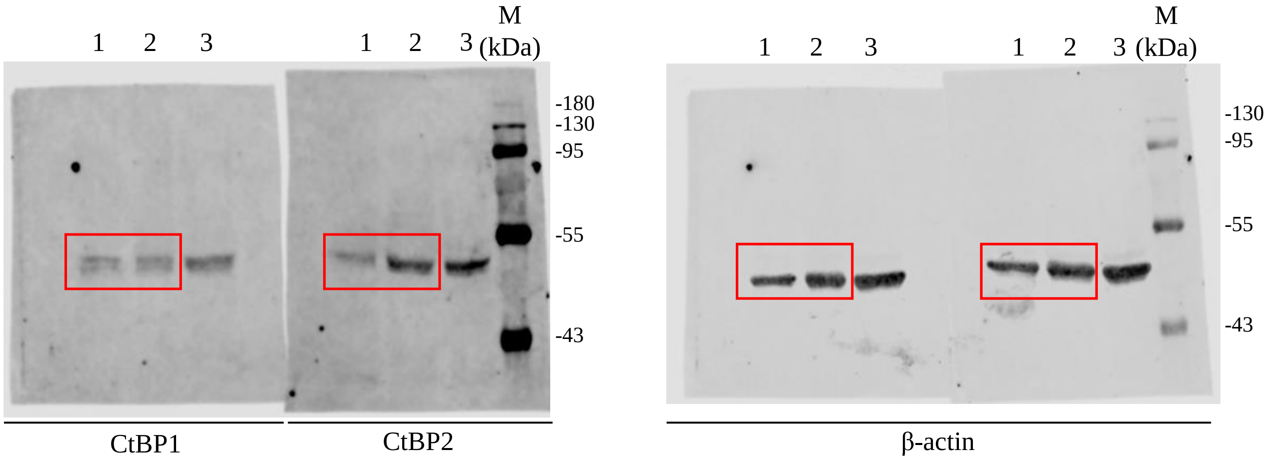

1= MCF102A; 2= MDA-MB231; 3= T47D (not included in this study)

	Densitometry signals		
	Sample		
Primary antibody	MCF102A	MDA-MB231	
anti-CtBP1	3	3.13	Secondary antibody: IRDye® 680RD Donkey anti-Mouse
anti-CtBP2	5.02	9.66	
anti-β-actin (CtBP1)	0.897	1.18	Secondary antibody: IRDye® 800CW Donkey anti-Rabbit
anti-β-actin (CtBP2)	1.18	1.48	

B

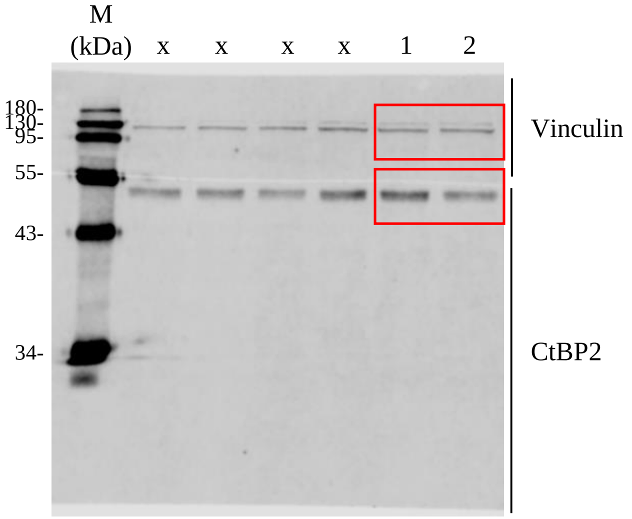

1= MDA-MB231 Scramble; 2= MDA-MB231 shCtBP2;
x=other shCtBP2 RNA clones not included in this study

	Densitometry signals		
	Sample		
Primary antibody	Scramble	shCtBP2	
anti-vinculin	2.05	2.13	Secondary antibody: IRDye® 680RD Donkey anti-Mouse
anti-CtBP2	4.33	2.54	
