# Supplementary material for: Transcriptomics and Metabolomics Integration Reveals Redox-Dependent Metabolic Rewiring in Breast Cancer Cells
Source: Cancers (Basel). 2021 Oct 9;13(20):5058. doi: 10.3390/cancers13205058 (PMC8534001; doi:10.3390/cancers13205058)
Supplement: Supplementary file 1 [file cancers-13-05058-s001.zip › cancers-1387848-SI/File S7.pdf]

Mass Hunter PCDL Single Search Results

Compound Name	Formula	Mass	Anion	Cation	RT (min)	CAS	ChemSpider	HMP	KEGG	LMP	IUPAC Name	Spectra
(S)-1-Pyrroline-5-carboxylate	C5H7NO2	113.04768			1.899				C03912			0
10-Formyltetrahydrofolate	C20H23N7O7	473.16590			12.696			HMDB00972	C00234			0
3-Phosphoglyceric acid	C3H7O7P	185.99294			3.109			HMDB00807	C00597			0
4-Hydroxyproline	C5H9NO3	131.05824			3.251			HMDB00725	C01157			0
5,10-Methenyltetrahydrofolate	C20H22N7O6	456.16316			1.440			HMDB01533	C00445			0
5,10-Methylene-THF	C20H23N7O6	457.17098			1.461			HMDB02365	C00143			0
5-Formimino-THF	C20H24N8O6	472.18188			1.745				C00664			0
5-Methyltetrahydrofolate	C20H25N7O6	459.18663			1.274				C00440			0
5'-Methylthioadenosine	C11H15N5O3S	297.08956			9.684			HMDB01173	C00170			0
6-Phosphogluconic acid	C6H13O10P	276.02463			6.934			HMDB01316	C00345			0
Acetoacetic acid	C4H6O3	102.03169			2.528			HMDB00060	C00164	LMFA01060003		0
Acetyl-CoA	C23H38N7O17P3S	809.12577			1.272			HMDB01206	C00024			0
Adenine	C5H5N5	135.05450			2.530				C00147			0
Adenosine	C10H13N5O4	267.09675			2.277			HMDB00050	C00212			0
Adenosine monophosphate	C10H14N5O7P	347.06308			2.623			HMDB00045	C00020			0
Adenosine triphosphate	C10H16N5O13P3	506.99575			15.219			HMDB00538	C00002			0
ADP	C10H15N5O10P2	427.02941			13.011			HMDB01341	C00008			0
AICAR	C9H15N4O8P	338.06275			2.289			HMDB01517	C04647			0
Allocystathionine	C7H14N2O4S	222.06743			1.749			HMDB00455	C02291			0
Allysine	C6H11NO3	145.07389			3.491			HMDB01263	C04076			0
alpha-D-Glucose 6-phosphate	C6H13O9P	260.02972			3.470				C00668			0
Beta-Alanine	C3H7NO2	89.04768			3.092			HMDB00056	C00099			0
CDP	C9H15N3O11P2	403.01818			2.382			HMDB01546	C00112			0
cis-Aconitic acid	C6H6O6	174.01644			4.015			HMDB00072	C00417			0
Citric acid	C6H8O7	192.02700			2.738			HMDB00094	C00158			0

Compound Name	Formula	Mass	Anion	Cation	RT (min)	CAS	ChemSpider	HMP	KEGG	LMP	IUPAC Name	Spectra
Citrulline	C6H13N3O3	175.09569			1.336			HMDB00904	C00327			0
Creatine	C4H9N3O2	131.06948			1.212			HMDB00064	C00300			0
Crotonoyl-CoA	C25H40N7O17P3S	835.14142			4.099			HMDB02009	C00877			0
Cyclic AMP	C10H12N5O6P	329.05252			9.559				C00575			0
Cys-Gly	C5H10N2O3S	178.04121			2.300			HMDB28775	C01419			0
D-1-Piperideine-2-carboxylic acid	C6H9NO2	127.06333			7.823			HMDB01084	C04092			0
dCTP	C9H16N3O13P3	466.98960			14.351			HMDB00998	C00458			0
Deoxyadenosine triphosphate	C10H16N5O12P3	491.00083			14.915				C00131			0
Deoxycytidine	C9H13N3O4	227.09061			2.235			HMDB00014	C00881			0
Deoxyuridine triphosphate	C9H15N2O14P3	467.97361			4.057			HMDB01191	C00460			0
D-Glucose	C6H12O6	180.06339			1.233			HMDB00516	C00221			0
D-Glyceraldehyde 3-phosphate	C3H7O6P	169.99802			14.435				C00118			0
Dihydrofolic acid	C19H21N7O6	443.15533			1.397			HMDB01056	C00415			0
dTDP	C10H16N2O11P2	402.02293			1.463			HMDB01274	C00363			0
dUDP	C9H14N2O11P2	388.00728			1.849			HMDB01000	C01346			0
Farnesyl diphosphate	C15H28O7P2	382.13103			12.866			HMDB00961	C00448	LMPR0103010002		0
Folate	C19H19N7O6	441.13968			12.761			HMDB00121	C00504			0
Fructose 1,6-bisphosphate	C6H14O12P2	339.99605			14.935			HMDB60444	C05378			0
Fumaric acid	C4H4O4	116.01096			1.400			HMDB00134	C00122			0
Gamma-Glutamylcysteine	C8H14N2O5S	250.06234			2.403				C03646			0
Geranyl diphosphate	C10H20O7P2	314.06843			1.212			HMDB01285	C00341	LMPR0102010001		0
Geranylgeranyl diphosphate	C20H36O7P2	450.19363			1.777			HMDB60109	C00353	LMPR0104010001		0
Glucosamine 6-phosphate	C6H14NO8P	259.04570			3.030			HMDB01254	C00352			0
Glutathione	C10H17N3O6S	307.08381			12.908			HMDB00125	C00051			0
Glyceric acid 1,3-biphosphate	C3H8O10P2	265.95927			2.927			HMDB01270	C00236			0
Glycerol 3-phosphate	C3H9O6P	172.01367			4.015			HMDB00126	C00093			0
Glycine	C2H5NO2	75.03203			1.233			HMDB00123	C00037			0
Guanidoacetic acid	C3H7N3O2	117.05383			14.872			HMDB00128	C00581			0
Guanine	C5H5N5O	151.04941			1.544				C00242			0
Guanosine monophosphate	C10H14N5O8P	363.05800			9.309			HMDB01397	C00144			0
Hypotaurine	C2H7NO2S	109.01975			1.274			HMDB00965	C00519			0
Hypoxanthine	C5H4N4O	136.03851			1.838				C00262			0
Inosinic acid	C10H13N4O8P	348.04710			2.341			HMDB00175	C00130			0
L-1-Pyrroline-3-hydroxy-5-carboxylate	C5H7NO3	129.04259			3.030				C04281			0
L-4-Hydroxyglutamate semialdehyde	C5H9NO4	147.05316			1.315			HMDB06556	C05938			0
L-Acetylcarnitine	C9H17NO4	203.11576			13.180			HMDB00201	C02571			0
L-Alanine	C3H7NO2	89.04768			3.025			HMDB00161	C00041			0
L-Arginine	C6H14N4O2	174.11168			1.189				C00062			0
L-Asparagine	C4H8N2O3	132.05349			1.295			HMDB00168	C00152			0
L-Aspartic acid	C4H7NO4	133.03751			1.398			HMDB00191	C00049			0
L-Cysteine	C3H7NO2S	121.01975			8.429			HMDB00574	C00097			0
L-Glutamic acid	C5H9NO4	147.05316			1.315			HMDB00148	C00025			0
L-Glutamic acid 5-phosphate	C5H10NO7P	227.01949			3.051			HMDB01228	C03287			0
L-Glutamic gamma-semialdehyde	C5H9NO3	131.05824			3.429			HMDB02104	C01165			0

Compound Name	Formula	Mass	Anion	Cation	RT (min)	CAS	ChemSpider	HMP	KEGG	LMP	IUPAC Name	Spectra
L-Glutamine	C5H10N2O3	146.06914			1.233			HMDB00641	C00064			0
L-Homocysteine	C4H9NO2S	135.03540			14.239				C00155			0
L-Lactic acid	C3H6O3	90.03169			2.111			HMDB00190	C00186			0
L-Malic acid	C4H6O5	134.02152			2.340			HMDB00156	C00149			0
L-Methionine	C5H11NO2S	149.05105			1.588			HMDB00696	C00073			0
L-Proline	C5H9NO2	115.06333			1.358			HMDB00162	C00148			0
L-Serine	C3H7NO3	105.04259			1.233			HMDB00187	C00065			0
L-Threonine	C4H9NO3	119.05824			1.233			HMDB00167	C00188			0
L-Tryptophan	C11H12N2O2	204.08988			9.987			HMDB00929	C00078			0
L-Tyrosine	C9H11NO3	181.07389			2.258			HMDB00158	C00082			0
Mevalonate-P	C6H13O7P	228.03989			2.181			HMDB01343	C01107	LMFA01050415		0
Mevalonate-PP	C6H14O10P2	308.00622			3.011			HMDB01090	C01143	LMFA01050416		0
Mevalonic acid	C6H12O4	148.07356			1.230			HMDB59629	C00418	LMFA01050352		0
N6-Acetyl-L-lysine	C8H16N2O3	188.11609			1.524			HMDB00206	C02727			0
N-Acetyl-alpha-D-glucosamine 6-phosphate	C8H16NO9P	301.05627			3.887				C04501			0
NAD	C21H27N7O14P2	663.10912			6.170				C00003			0
NADH	C21H29N7O14P2	665.12477			13.577			HMDB01487	C00004			0
NADP	C21H29N7O17P3	744.08328			12.970			HMDB00217	C00006			0
NADPH	C21H30N7O17P3	745.09110			15.292				C00005			0
Ornithine	C5H12N2O2	132.08988			1.170			HMDB00214	C00077			0
Oxidized glutathione	C20H32N6O12S2	612.15196			3.219			HMDB03337	C00127			0
Oxoglutaric acid	C5H6O5	146.02152			2.277			HMDB00208	C00026			0
Phosphoribosyl pyrophosphate	C5H13O14P3	389.95181			2.509			HMDB00280	C00119			0
Pyruvic acid	C3H4O3	88.01604			2.381			HMDB00243	C00022	LMFA01060077		0
Reduced riboflavin	C17H22N4O6	378.15393			12.991			HMDB01557	C01007			0
Riboflavin	C17H20N4O6	376.13828			1.337			HMDB00244	C00255			0
S-Adenosylhomocysteine	C14H20N6O5S	384.12159			1.316			HMDB00939	C00021			0
S-Adenosyl-L-methionine	C15H22N6O5S	398.13724			1.588				C00019			0
S-Adenosylmethioninamine	C14H23N6O3S	355.15523			1.839			HMDB00988	C01137			0
SAICAR	C13H19N4O12P	454.07371			9.093			HMDB00797	C04823			0
Sedoheptulose 7-phosphate	C7H15O10P	290.04028			3.911				C05382			0
Succinic acid	C4H6O4	118.02661			2.675			HMDB00254	C00042	LMFA01170043		0
Succinic acid semialdehyde	C4H6O3	102.03169			2.590			HMDB01259	C00232	LMFA06000118		0
Taurine	C2H7NO3S	125.01466			1.315			HMDB00251	C00245			0
Tetrahydrofolate	C19H23N7O6	445.17098			14.791				C00101			0
Thymidine	C10H14N2O5	242.09027			15.230			HMDB00273	C00214			0
Uric acid	C5H4N4O3	168.02834			2.389			HMDB00289	C00366			0
Uridine 5'-diphosphate	C9H14N2O12P2	404.00220			13.598			HMDB00295	C00015			0
Uridine triphosphate	C9H15N2O15P3	483.96853			13.848			HMDB00285	C00075			0
Xylulose 5-phosphate	C5H11O8P	230.01915			6.964			HMDB00868	C00231			0
