# Supplementary figures and images for: Transcriptomics and Metabolomics Integration Reveals Redox-Dependent Metabolic Rewiring in Breast Cancer Cells
Source: Cancers (Basel). 2021 Oct 9;13(20):5058. doi: 10.3390/cancers13205058 (PMC8534001; doi:10.3390/cancers13205058)

**A**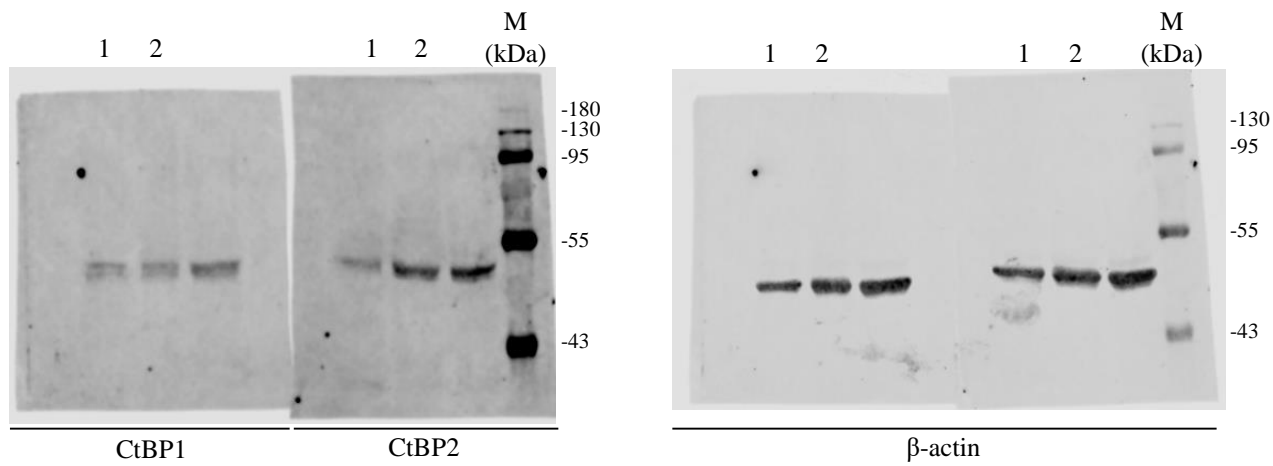**B**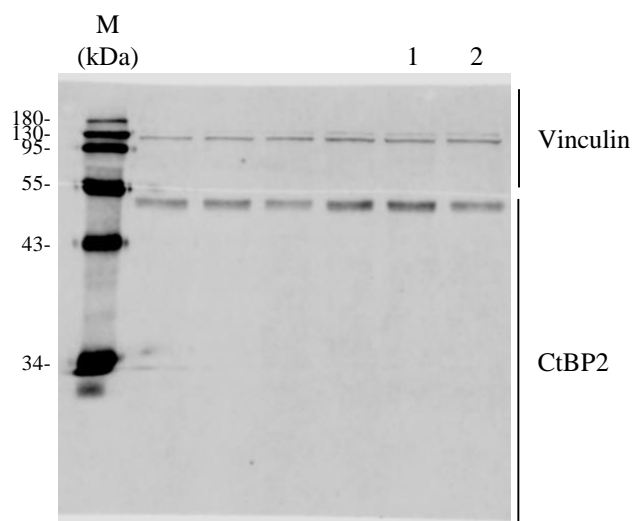

Supplement: Supplementary file 1 [file cancers-13-05058-s001.zip › cancers-1387848-SI/cancers-1387848-WB.pdf]
